# An engineered ROS-responsive cascade nanoplatform delays Alzheimer's disease progression via Nrf2/GPX4-mediated microglial functional reprogramming

**DOI:** 10.1016/j.mtbio.2026.103055

**Published:** 2026-03-27

**Authors:** Yang Yu, Jun-jie Yu, Shuai-wen Ding, Ge Zhang, Yang Liu, Rui-bo Guo, Juan Zang, Xiang-xuan Zhao, Xue-tao Li, Liang Kong

**Affiliations:** a Key Laboratory of Ministry of Education for Traditional Chinese Medicine Viscera-State Theory and Applications, Liaoning University of Traditional Chinese Medicine, Shenyang, 110847, China; b College of Pharmacy, Liaoning University of Traditional Chinese Medicine, Dalian, 116600, China; c Department of Biomedical Engineering, Dalian University of Technology, Dalian, 116024, China; d College of Laboratory Animal Medicine, Liaoning University of Traditional Chinese Medicine, Shenyang, 110847, China; e Shenyang Key Laboratory of Chinese Medicine Targeted Delivery, Shenyang, 110148, China

**Keywords:** Alzheimer's disease, Microglial, Ferroptosis, Nrf2/GPX4

## Abstract

Alzheimer's disease (AD) is driven by a self-amplifying pathological network in which microglia-mediated neuroinflammation, oxidative stress, and cerebral iron dyshomeostasis are tightly interconnected. Here, we report a ROS-responsive, cascade-targeted nanoplatform (KMAI@NPs) engineered to intervene at this microglia-centered regulatory hub. The nanoplatform integrates an acetylsalicylic acid-modified dextran self-assembly core with PEGylated peptide modules for cascade targeting. By systematically optimizing the dextran molecular weight and acetylsalicylic acid grafting ratio, the self-assembly behavior and in vivo stability of the nanoplatform were rationally tuned. KMAI@NPs efficiently penetrate the blood-brain barrier, exhibit prolonged circulation, and selectively target activated microglia. Mechanistically, KMAI@NPs regulate microglial polarization and inhibit ferroptosis. In particular, given that M2-polarized microglia are more susceptible to ferroptosis, KMAI@NPs further protect these beneficial cells from ferroptotic injury through activation of the Nrf2/GPX4 axis, thereby preserving their anti-inflammatory and neuroprotective functions under inflammatory and iron-overload conditions. In APP/PS1 transgenic mice, KMAI@NPs markedly alleviate neuroinflammation, iron overload, amyloid pathology, and neuronal ultrastructural damage, resulting in significant cognitive improvement. This work establishes a microglia-centered, multitarget nanotherapeutic strategy that enables coordinated regulation of neuroinflammation, oxidative stress, and iron dyshomeostasis in AD.

## Graphical abstract

**Figure:**
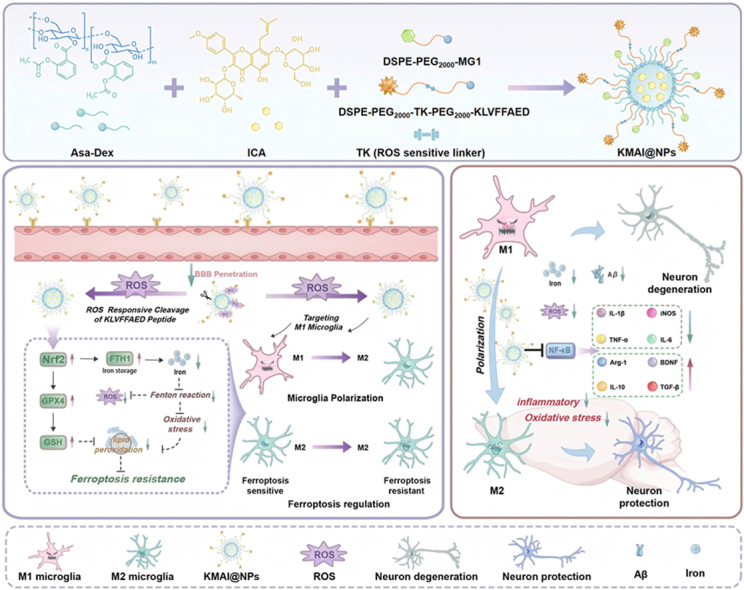

## 1 Introduction

Alzheimer's disease (AD) is a progressive neurodegenerative disorder characterized by cognitive decline and memory impairment [1]. AD progression is driven by multiple coupled pathological cascades, limiting the sustained efficacy of single-target therapeutic strategies. Consequently, current research has shifted from single-target interventions toward identifying and modulating key pathological hubs that act as central amplifiers of disease progression [[2], [3], [4]]. Within this intricate network, microglia, the resident immune cells of the central nervous system, are now recognized as a pivotal regulatory nexus linking neuroinflammation, oxidative stress, and neurodegeneration [[5], [6], [7], [8]]. During AD progression, microglia undergo a transition from a homeostatic surveillance state to a chronically activated phenotype, accompanied by profound functional dysregulation and a pronounced shift toward a pro-inflammatory (M1-like) profile. Persistently activated microglia continuously release ROS and pro-inflammatory cytokines, thereby amplifying neuroinflammatory signaling and exacerbating neuronal injury. In contrast, the anti-inflammatory and neuroprotective (M2-like) microglial phenotype is markedly suppressed in AD brains [[9], [10], [11]]. Disruption of this dynamic phenotypic balance is increasingly understood to drive a self-reinforcing loop linking neuroinflammation, neuronal damage, and neurodegenerative progression. Accordingly, functional reprogramming of microglia, rather than indiscriminate suppression of inflammatory signals, has emerged as a promising therapeutic paradigm for AD [12].

Recent evidence has further demonstrated that aberrant microglial activation and cerebral iron dyshomeostasis in AD are tightly coupled and mutually reinforcing, forming a self-sustaining pathological feedback loop [13]. Chronic microglial activation is accompanied by dysregulated iron uptake, storage, and export, leading to progressive intracellular iron accumulation. Excess labile iron catalyzes continuous ROS production via Fenton reactions, thereby aggravating oxidative stress and further stabilizing a pro-inflammatory microglial state. Under sustained iron overload and lipid peroxidation, microglia become particularly susceptible to ferroptosis, an iron-dependent form of regulated cell death characterized by glutathione depletion and impaired glutathione peroxidase 4 (GPX4) activity. Ferroptotic microglia may subsequently release damage-associated molecular patterns, activating surrounding glial cells and amplifying neuroinflammatory signaling [[14], [15], [16]]. Collectively, microglia act not only as central regulators of the crosstalk between iron metabolism and inflammation, but also as vulnerable cellular targets within the cycle linking iron overload, ferroptosis, and inflammation in AD.

In this context, natural products with pleiotropic regulatory properties represent important molecular candidates for therapeutic intervention. Icariin (ICA), a prenylated flavonol glycoside, exhibits potent antioxidant activity, and its polyhydroxylated flavonoid structure confers potential metal-ion chelating capacity, rendering it advantageous for regulating oxidative stress and iron homeostasis [[17], [18], [19]]. Meanwhile, the classical anti-inflammatory drug acetylsalicylic acid (Asa) effectively attenuates neuroinflammation through modulation of inflammation-related signaling pathways [[20], [21], [22]]. From a mechanistic perspective, ICA and Asa are therefore expected to exert complementary regulatory effects on oxidative stress, inflammation, and iron metabolism. However, the therapeutic application of such multi-target agents is severely constrained by poor blood-brain barrier (BBB) penetration, insufficient cellular specificity, and rapid systemic clearance, underscoring the critical need for precise co-delivery strategies within the central nervous system [[23], [24], [25]].

**Scheme 1:**
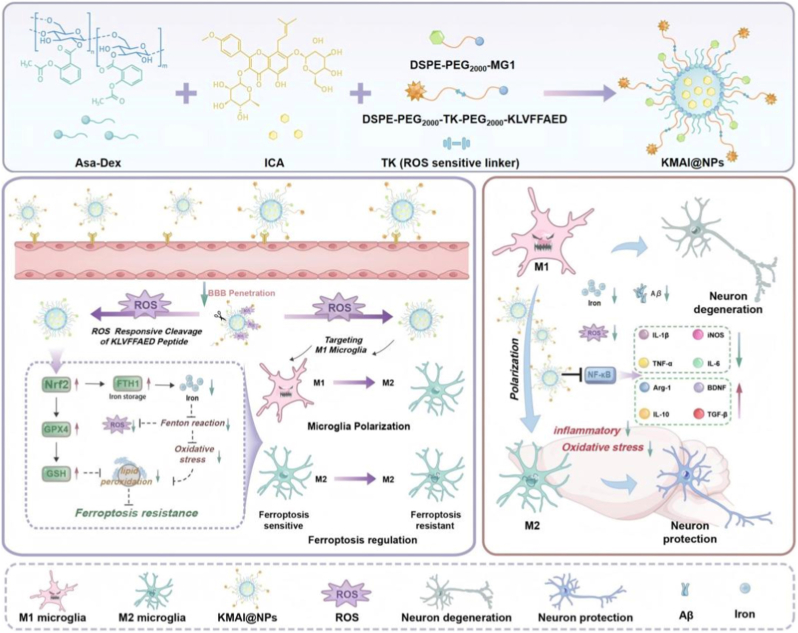
Schematic illustration of the construction and anti-AD mechanisms of KMAI@NPs

Engineered nanocarrier systems provide a promising platform for coordinated multi-target drug delivery by prolonging systemic circulation, enhancing brain accumulation, and reducing off-target toxicity [[26], [27], [28], [29], [30]]. Therefore, in this study, as shown in (Scheme 1), we developed a ROS-responsive, cascade-targeted nanoplatform (KMAI@NPs) designed to leverage microglia as a central regulatory node. The nanoplatform was rationally engineered using a dextran-based acetylsalicylic acid prodrug carrier for efficient encapsulation of icariin, enabling stable co-loading and synchronized delivery of the two agents. To achieve precise targeting, the nanoparticle surface was further functionalized with a dual-peptide architecture. The primary targeting peptide KLVFFAED (KLVFF) facilitates BBB translocation, while ROS-responsive cleavage in the pathological microenvironment exposes the secondary targeting peptide MG1, enabling selective recognition of activated microglia. In vitro and in vivo characterization demonstrated that KMAI@NPs exhibit a uniform spherical morphology, an appropriate nanoscale size, favorable dispersibility, and excellent circulation stability and biosafety. The nanoplatform efficiently traverses the BBB and, through a ROS-responsive activation mechanism, enables selective targeting of activated (M1-like) microglia. In APP/PS1 transgenic mice, KMAI@NPs produced markedly superior therapeutic outcomes compared with free ICA/Asa combinations or non-targeted formulations, including attenuation of cerebral oxidative stress and neuroinflammation, reduction of amyloid-β deposition, and preservation of neuronal mitochondrial ultrastructure and dendritic integrity. In parallel, KMAI@NPs modulated cerebral iron homeostasis, as evidenced by reduced pathological iron accumulation and upregulated expression of the iron-storage protein FTH1. Further analyses indicated that KMAI@NPs reprogram microglial functional states by promoting a shift from a pro-inflammatory (M1-like) phenotype toward an anti-inflammatory (M2-like) profile, accompanied by engagement of the Nrf2/GPX4-associated signaling axis and mitigation of ferroptosis-related molecular features. These coordinated effects are consistent with the observed improvement in cognitive performance in AD model mice. Collectively, this study presents a cascade-targeted, microglia-centered nanotherapeutic strategy that enables coordinated modulation of inflammation, oxidative stress, and iron dysregulation, offering a promising framework for intervening in the tightly coupled pathogenic network underlying AD.

## 2 Results and discussion

### 2.1 Preparation and characterization of KMAI@NPs

**Scheme 2:**
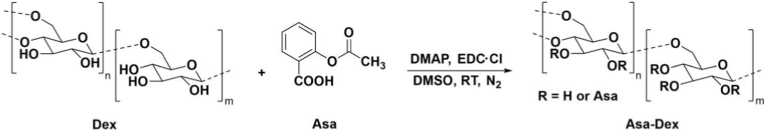
Schematic representation of the synthetic route.

**Fig. 1:**
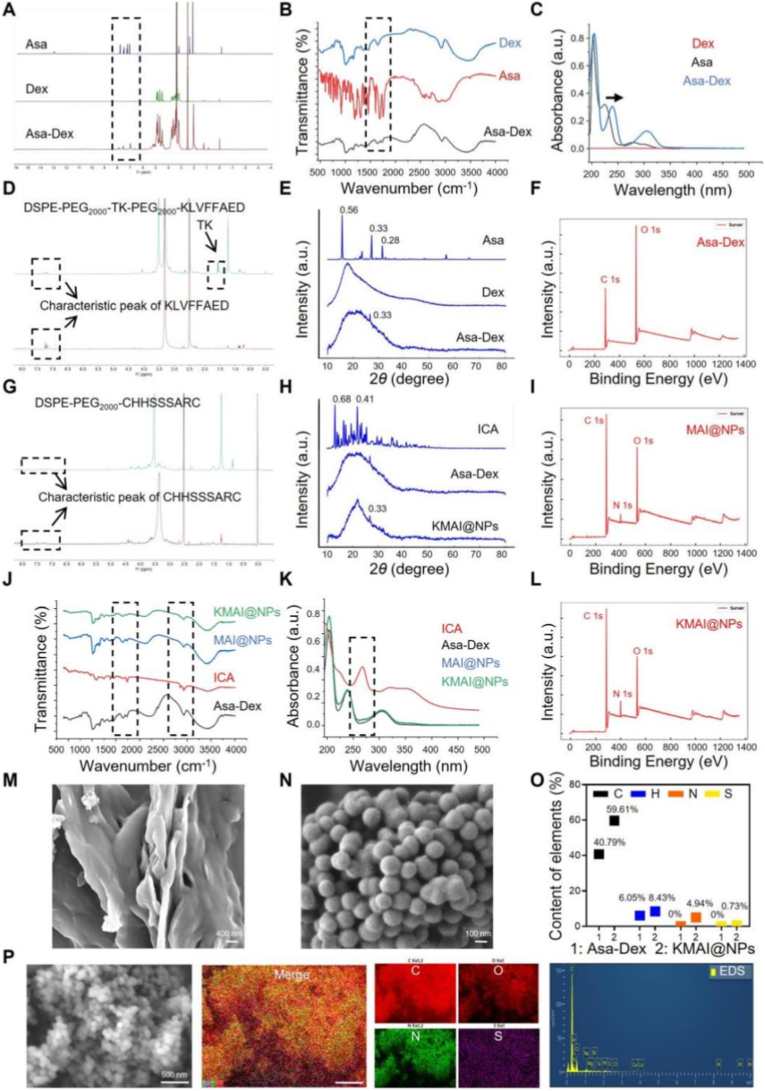
Synthesis and physicochemical characterization of KMAI@NPs. (**A**) ^1^H NMR spectra of Asa, Dex, and Asa-Dex solubilized in DMSO-*d*_*6*_. (**B**) FTIR analysis of Asa, Dex, and Asa-Dex. (**C**) UV-spectra of Asa, Dex, and Asa-Dex. (**D**) ^1^H NMR spectra of DSPE-PEG_2000_-TK-PEG_2000_-KLVFFAED solubilized in DMSO-*d*_*6*_. (**E**) X-ray diffraction (XRD) patterns of Asa, Dex, and Asa-Dex. (**F**) X-ray photoelectron spectroscopy (XPS) survey spectrum of Asa-Dex, confirming elemental composition. (**G**) ^1^H NMR spectra of DSPE-PEG_2000_-MG1 solubilized in DMSO-*d*_*6*_. (**H**) XRD patterns of ICA, Asa-Dex, and KMAI@NPs. (**I**) XPS survey spectrum of MAI@NPs. (**J**) FTIR analysis of different kinds of formulations including ICA, Asa-Dex, MAI@NPs and KMAI@NPs. (**K**) UV-spectra of ICA, Asa-Dex, MAI@NPs and KMAI@NPs. (**L**) XPS survey spectrum of KMAI@NPs. (**M**) SEM images of Dex. Scale bar: 400 nm. (**N**) SEM images of KMAI@NPs. Scale bar: 100 nm. (**O**) Elemental composition analysis of Asa-Dex and KMAI@NPs. (**P**) SEM surface morphology and EDS element mapping of KMAI@NPs. Scale bar: 500 nm.

In this study, biocompatible dextran (Dex) was employed as the carrier backbone due to its favorable biological safety and ease of chemical modification [31]. Asa was covalently grafted onto Dex via an esterification reaction to yield an amphiphilic conjugate, Asa-Dex (Scheme 2). In the ^1^H NMR spectrum (Fig. 1A), characteristic signals corresponding to the methyl group of Asa (*δ* 2.3 ppm), aromatic protons (*δ* 6.9–8.0 ppm), and the Dex backbone protons (*δ* 3.0–5.0 ppm) were simultaneously observed, confirming the successful conjugation of Asa to Dex. FTIR spectra further supported ester bond formation, as evidenced by the disappearance of the characteristic carboxyl stretching band of free Asa (1700–1750 cm^−1^) and the emergence of a new ester carbonyl vibration band at 1735–1750 cm^−1^ (Fig. 1B). UV-vis absorption spectra revealed that Asa-Dex exhibited a markedly different absorption profile from free Asa in the 200–350 nm range (Fig. 1C), further verifying successful conjugate formation.

The self-assembly properties of Asa-Dex conjugates synthesized under different conditions were systematically evaluated, as summarized in Supplementary Table S1. The Asa conjugation content was quantified by UV-vis spectroscopy using a calibration curve of free Asa, while the critical aggregation concentration (CAC) was determined by a fluorescence probe method. When Dex with a relatively low molecular weight of 1000 Da was used, no distinct CAC could be detected, indicating that the conjugate failed to form stable micellar assemblies in aqueous solution. This behavior is attributable to the insufficient hydrophilic chain length of Dex, which is unable to effectively stabilize amphiphilic aggregates, even at a relatively high Asa conjugation content of 12.2%. In contrast, increasing the Dex molecular weight to 2000 Da enabled partial self-assembly, yielding a relatively high CAC of 0.879 mg/mL. Further increasing the Dex molecular weight to 6000 Da markedly enhanced the self-assembly capability, as evidenced by a substantial reduction in CAC to 0.0126 mg/mL, observed at an Asa conjugation content of 7.7%.

For Asa-Dex conjugates based on Dex with a molecular weight of 6000 Da, a clear dependence of CAC on Asa conjugation content was observed. At low conjugation levels of 0.51–0.63%, the conjugates exhibited relatively high CAC values ranging from 0.539 to 0.277 mg/mL, suggesting weak amphiphilicity. As the Asa conjugation content increased to an intermediate range of 2.3–3.6%, the CAC gradually decreased to 0.196–0.106 mg/mL, indicating enhanced self-assembly driven by strengthened hydrophobic interactions. Notably, the lowest CAC values were achieved at moderate to high conjugation levels of 7.7–8.5%, reaching 0.0126–0.0937 mg/mL. However, a further increase in Asa conjugation content to 10.3% resulted in a pronounced rise in CAC to 0.691 mg/mL, indicating that excessive hydrophobic modification can compromise effective self-assembly.

To construct a cascade-targeted delivery system, two types of functional targeting molecules were synthesized and characterized. The ^1^H NMR spectrum of the first-level targeting molecule, DSPE-PEG_2000_-TK-PEG_2000_-KLVFFAED, displayed characteristic signals corresponding to the ROS-sensitive thioacetal ketone (TK) linkage (*δ* 1.5–1.6 and 2.6–2.7 ppm), PEG segments (*δ* 3.5 ppm), and KLVFF peptide (*δ* 7.1–7.5 ppm), confirming successful construction of the ROS-responsive primary targeting module (Fig. 1D). The ^1^H NMR spectrum of the secondary targeting molecule, DSPE-PEG_2000_-MG1, exhibited characteristic MG1 peptide signals in the range of *δ* 7.0–8.5 ppm (Fig. 1G).

Subsequently, the hydrophobic drug ICA was encapsulated into the Asa-Dex core via a self-assembly strategy, together with the incorporation of the targeting modules, yielding the final nanoplatform KMAI@NPs. FTIR and UV-vis analyses demonstrated that the characteristic absorption peaks of ICA were markedly attenuated or disappeared in both MAI@NPs and KMAI@NPs (Fig. 1J and K), indicating efficient drug encapsulation rather than simple physical adsorption, where MAI@NPs represent MG1 single-ligand modified nanoparticles, and KMAI@NPs denote dual-targeted nanoparticles functionalized with both KLVFF and MG1 peptides. Encapsulation efficiency (EE%) analysis further supported these observations, with an initial EE exceeding 90% and remaining at approximately 80% after one week of storage (Supplementary, Table S2), demonstrating favorable loading stability. XRD analysis provided direct evidence of the physical state of the drug (Fig. 1E–H), while free ICA and Asa exhibited sharp crystalline diffraction peaks, no characteristic ICA/Asa diffraction signals were observed in KMAI@NPs, indicating that ICA/Asa was dispersed in an amorphous state within the nanocarrier, which is advantageous for improving solubility and delivery stability.

To further verify the successful assembly of the cascade-targeting architecture, XPS and elemental analyses were performed. Asa-Dex exhibited only C 1s and O 1s signals (Fig. 1F), whereas MAI@NPs displayed an additional N 1s signal following peptide modification (Fig. 1I). In the fully constructed KMAI@NPs, the N 1s signal intensity was further enhanced (Fig. 1L). Consistently, elemental analysis revealed the presence of N and S elements in KMAI@NPs but not in Asa-Dex (Fig. 1O), where nitrogen originates from the peptide ligands and sulfur from the thioacetal ketone linkage. These results collectively confirm the successful integration of the functional targeting modules onto the nanoparticle surface.

Morphological characterization further validated nanoparticle formation at the microscopic level. Scanning electron microscopy (SEM) images showed that KMAI@NPs possessed a uniform spherical morphology with narrow size distribution (Fig. 1N), in sharp contrast to the amorphous aggregated structure of pristine Dex (Fig. 1M). EDS elemental mapping demonstrated homogeneous distribution of C, O, N, and S elements throughout the nanoparticles (Fig. 1P), further supporting the structural uniformity of the nanoplatform.

**Fig. 2:**
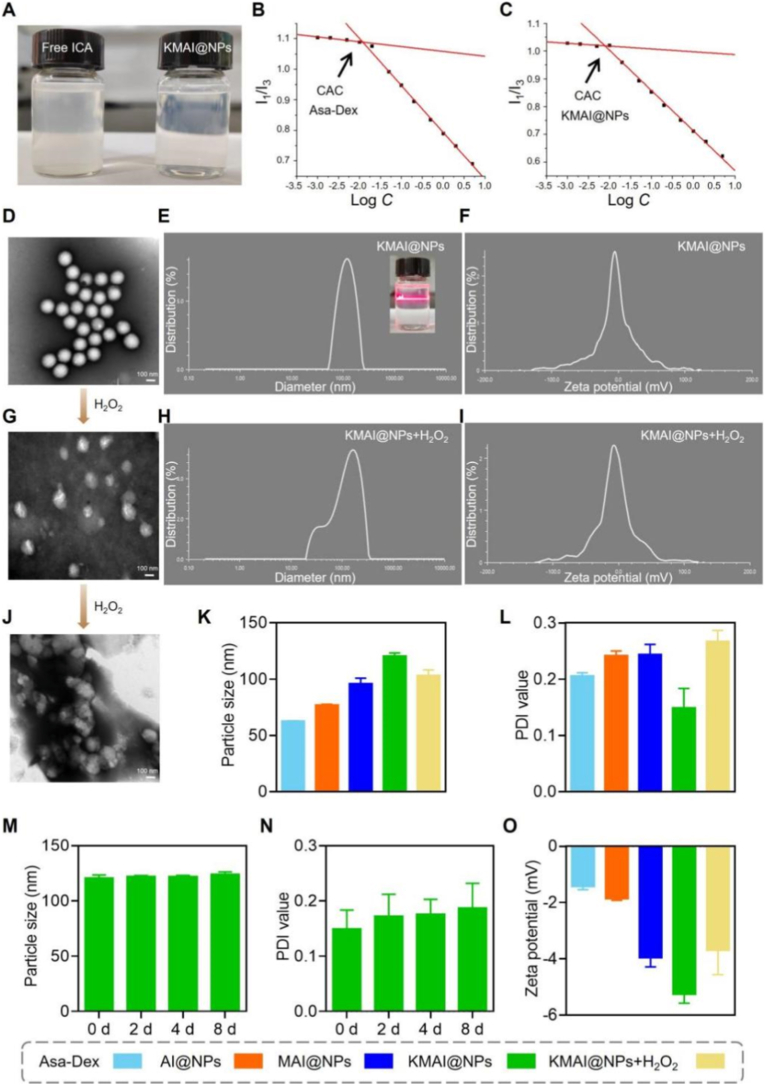
Formation, ROS-responsiveness, and colloidal stability of KMAI@NPs. (**A**) Photographs of ICA aqueous solution and KMAI@NPs. (**B**) CAC of Asa-Dex, 0.0126 mg/mL (**C**) CAC of KMAI@NPs, 0.00796 mg/mL (**D**) TEM image of KMAI@NPs, showing uniform spherical nanostructures. Scale bar: 100 nm. (**E**) Hydrodynamic diameter distribution of KMAI@NPs measured by DLS. (**F**) Zeta potential distribution of KMAI@NPs. (**G**) TEM image of KMAI@NPs after incubation with PBS containing H_2_O_2_ for 1 h. Scale bar: 100 nm. (**H**) Hydrodynamic diameter distribution of KMAI@NPs after H_2_O_2_ treatment. (**I**) Zeta potential distribution of KMAI@NPs after H_2_O_2_ treatment. (**J**) TEM image of KMAI@NPs after incubation with PBS containing H_2_O_2_ for 24 h. Scale bar: 100 nm. (**K**) Quantitative comparison of particle size among Asa-Dex, AI@NPs, MAI@NPs, KMAI@NPs, and KMAI@NPs + H_2_O_2_. (**L**) Quantitative comparison of polydispersity index (PDI) values among different formulations. (**M**) Time-dependent stability analysis of KMAI@NPs particle size during storage at 4 °C for up to 8 days. (**N**) Time-dependent changes in PDI of KMAI@NPs during storage at 4 °C. (**O**) Quantitative comparison of zeta potential distribution among Asa-Dex, AI@NPs, MAI@NPs, KMAI@NPs, and KMAI@NPs + H_2_O_2_.

Following structural construction, the key physicochemical properties of KMAI@NPs were systematically evaluated. KMAI@NPs exhibited excellent aqueous dispersibility, forming a clear and transparent solution (Fig. 2A), whereas free ICA rapidly precipitated in water, indicating that nanoparticle self-assembly markedly enhanced the solubility of the hydrophobic drug. Pyrene fluorescence probe analysis revealed a CAC of 0.00796 mg/mL for KMAI@NPs (Fig. 2C), which is substantially lower than the dilution threshold under physiological blood circulation conditions (≈0.5 mg/mL), suggesting that the self-assembled structure remains stable even under highly diluted environments [32].

Transmission electron microscopy (TEM) observations showed that KMAI@NPs were monodisperse spherical nanoparticles (Fig. 2D). Dynamic light scattering (DLS) analysis indicated an average hydrodynamic diameter of (120.72 ± 2.71) nm, a low polydispersity index (PDI) of (0.149 ± 0.034), and a near-neutral zeta potential of −(5.27 ± 0.32) mV (Fig. 2E and F), reflecting favorable colloidal stability [33]. Compared with MAI@NPs lacking long-chain targeting ligands, KMAI@NPs exhibited a slightly larger particle size, consistent with surface functionalization (Supplementary, Fig. S1).

Upon incubation in an H_2_O_2_-simulated oxidative microenvironment, KMAI@NPs exhibited pronounced structural responsiveness. TEM images revealed the emergence of surface defects after short-term exposure, followed by progressive nanoparticle disassembly upon prolonged treatment (Fig. 2G–J). Correspondingly, DLS analysis showed a reduction in average particle size to (103.38 ± 4.95) nm, accompanied by changes in size distribution and zeta potential (Fig. 2H, I, K, L, O).

Further stability assessments demonstrated that KMAI@NPs maintained stable particle size and PDI values after storage at 4 °C for 8 days (Fig. 2M and N), indicating good storage stability. Taken together with their appropriate nanoscale size, near-neutral surface charge, extremely low CAC, and robust storage stability, these physicochemical characteristics support the potential of KMAI@NPs as a stable delivery system with favorable circulation properties in vivo.

Overall, the physicochemical characterization confirms that KMAI@NPs possesses stable drug loading, controllable ROS responsiveness, and colloidal properties compatible with in vivo applications.

### 2.2 Cellular uptake and cascade targeting performance

**Fig. 3:**
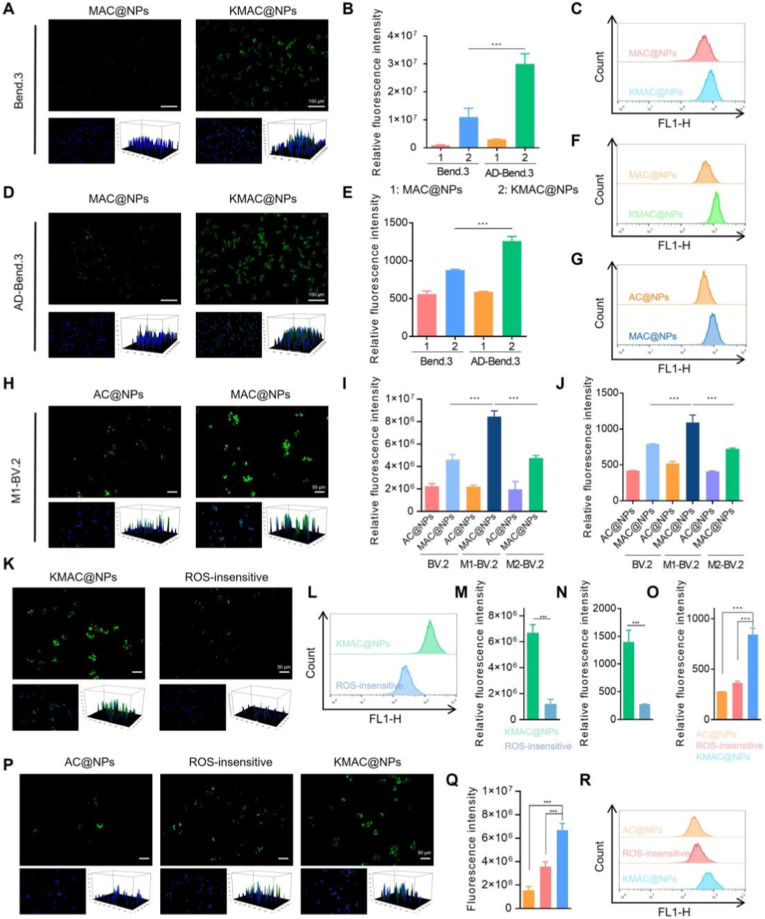
Evaluation of cascade microglial targeting and BBB penetration in vitro. (**A**) Cellular uptake in Bend.3 cells. Scale bars: 100 μm. (**B**) Semi-quantitative analysis of fluorescence intensity from microscopy images (A and D). (**C**) Analysis of cellular uptake of Bend.3 cells treated with MAC@NPs and KMAC@NPs by flow cytometry. (**D**) Cellular uptake in Aβ_1-42_-induced injured Bend.3 cells. Scale bars: 100 μm. (**E**) Quantitative analysis of fluorescence intensity from flow cytometry data (C and F). (**F**) Analysis of cellular uptake of AD-Bend.3 cells treated with MAC@NPs and KMAC@NPs by flow cytometry. (**G**) Flow cytometry analysis of M1-BV.2 cell uptake of AC@NPs and MAC@NPs. (**H**) Cellular uptake of non-targeted AC@NPs and MAC@NPs by M1-BV.2 cells (green: coumarin, blue: DAPI), Scale bar: 50 μm. (**I**) Semi-quantitative analysis of fluorescence intensity from microscopy images (H). (**J**) Quantitative analysis of fluorescence intensity from flow cytometry data (G). (**K**) Cellular uptake of ROS-insensitive KMAC@NPs and KMAC@NPs by M1-BV.2 cells, Scale bar: 50 μm. (**L**) Flow cytometry analysis of M1-BV.2 cell uptake of ROS-insensitive KMAC@NPs and KMAC@NPs. (**M**) Semi-quantitative analysis of fluorescence intensity from microscopy images (K). (**N**) Quantitative analysis of fluorescence intensity from flow cytometry data (L). (**O**) Quantitative analysis of fluorescence intensity of M1-BV.2 cells in the lower chamber of the Transwell system. (**P**) Cellular uptake by M1-BV.2 cells in the lower chamber of a Transwell system after treatment with AC@NPs, ROS-insensitive KMAC@NPs, and KMAC@NPs, Scale bar: 50 μm. (**Q**) Semi-quantitative analysis of fluorescence intensity from microscopy images (P). (**R**) Flow cytometry analysis of M1-BV.2 cell uptake in the lower chamber of a Transwell system with AC@NPs, ROS-insensitive KMAC@NPs, and KMAC@NPs. Results are reported as mean ± SD (n = 3, ∗∗∗*P* < 0.001). (For interpretation of the references to colour in this figure legend, the reader is referred to the Web version of this article.)

The BBB is a critical physiological barrier that prevents approximately 98% of small-molecule drugs and nearly all macromolecular therapeutics from entering brain tissue [34]. To evaluate the effect of KLVFF modification on the BBB-translocation capability of nanoparticles, cellular uptake studies were first performed using Bend.3 cells and Aβ_1-42_-induced injured Bend.3 cells (AD-Bend.3). Fluorescence microscopy and flow cytometry analyses demonstrated that coumarin-labeled dual-targeted nanoparticles (KMAC@NPs) exhibited significantly enhanced uptake in AD-Bend.3 cells compared with coumarin-labeled nanoparticles modified with MG1 alone (MAC@NPs) (Fig. 3A–F). Quantitative analysis confirmed that the fluorescence intensity of the KMAC@NPs group was markedly higher than that of the MAC@NPs group (∗∗∗*P* < 0.001), indicating that KLVFF effectively promotes nanoparticle interaction with BBB-associated cells under AD-mimicking conditions.

To further verify whether KMAC@NPs undergo ROS-responsive shedding of the outer KLVFF peptide under oxidative stress, a ROS-insensitive control nanoparticle was constructed by directly conjugating KLVFF to DSPE-PEG_4000_, which lacks the TK structure required for ROS sensitivity. A Förster resonance energy transfer (FRET) based in vitro assay was employed to evaluate KLVFF cleavage [35]. Specifically, Dabcyl-labeled KLVFF peptides and FITC-labeled MG1 peptides were simultaneously incorporated into KMAI@NPs. In the intact state, FITC fluorescence was quenched due to FRET. Upon exposure to pathologically relevant concentrations of H_2_O_2_, KMAI@NPs exhibited a pronounced recovery of FITC fluorescence compared with the ROS-insensitive control (Supplementary, Fig. S2), indicating that the nanoplatform rapidly responds to ROS stimulation and undergoes shedding of the outer KLVFF peptide layer.

Given that the targeting function of MG1 depends on its sufficient exposure, the microglia-targeting capability of KMAC@NPs following ROS activation was subsequently evaluated. BV.2 microglial cells were successfully polarized into M1 and M2 phenotypes using LPS + FAC or IL-4 treatment, respectively, as evidenced by significant upregulation of the M1 marker iNOS and the M2 marker Arg-1 (Supplementary, Fig. S3). Further cellular uptake studies revealed that in M1-polarized BV.2 cells, MAC@NPs were internalized significantly more efficiently than AC@NPs lacking MG1 modification (Fig. 3G–J), whereas the difference in uptake between AC@NPs and MAC@NPs was markedly reduced in resting or M2-polarized BV.2 cells (Supplementary, Fig. S4). Quantitative analysis demonstrated a pronounced enhancement of MAC@NPs uptake in M1 microglia (∗∗∗*P* < 0.001), consistent with the selective recognition of the M1 phenotype by the MG1 peptide.

On this basis, the influence of ROS responsiveness on M1-specific uptake was further examined. M1-polarized BV.2 cells were incubated with coumarin-labeled KMAC@NPs or ROS-insensitive KMAC@NPs. Both fluorescence microscopy and flow cytometry analyses revealed that KMAC@NPs were taken up by M1 microglia to a significantly greater extent than the ROS-insensitive control nanoparticles (Fig. 3K–N, ∗∗∗*P* < 0.001), indicating that ROS-triggered shedding of KLVFF facilitates MG1 exposure and enhances recognition by inflammatory microglia.

Finally, a Transwell migration model was employed to further assess the ability of nanoparticles to cross the BBB and subsequently target M1 microglia. Coumarin-labeled AC@NPs, ROS-insensitive KMAC@NPs, and KMAC@NPs were added to the upper chamber containing AD-Bend.3 cells, while M1-polarized BV.2 cells were cultured in the lower chamber. After 12 h of incubation, fluorescence microscopy and flow cytometry analyses demonstrated that uptake of KMAC@NPs by M1 microglia in the lower chamber was significantly higher than that observed for the other two formulations (Fig. 3O–R, ∗∗∗*P* < 0.001). These results indicate that KLVFF modification facilitates BBB traversal, and that subsequent ROS-triggered shedding of the outer KLVFF layer exposes the MG1 peptide, thereby promoting efficient uptake by inflammatory microglia.

Collectively, these findings demonstrate that KMAC@NPs achieve effective cascade targeting by sequentially enabling BBB penetration and inflammatory microglia-specific recognition, thereby facilitating precise delivery to M1 microglia under AD-associated pathological conditions.

### 2.3 In vivo circulation, biodistribution, and biosafety evaluation

To investigate the in vivo circulation behavior and brain-targeting performance of the nanoplatform, ICA was replaced with the near-infrared fluorescent probe DiR for real-time imaging studies. DiR-loaded nanoparticles without peptide modification were denoted as AD@NPs, nanoparticles modified with a single MG1 peptide were denoted as MAD@NPs, and nanoparticles modified with both KLVFF and MG1 peptides were denoted as KMAD@NPs. Whole-body fluorescence imaging was employed to monitor the biodistribution and accumulation of different formulations in AD model mice following intravenous administration.

**Fig. 4:**
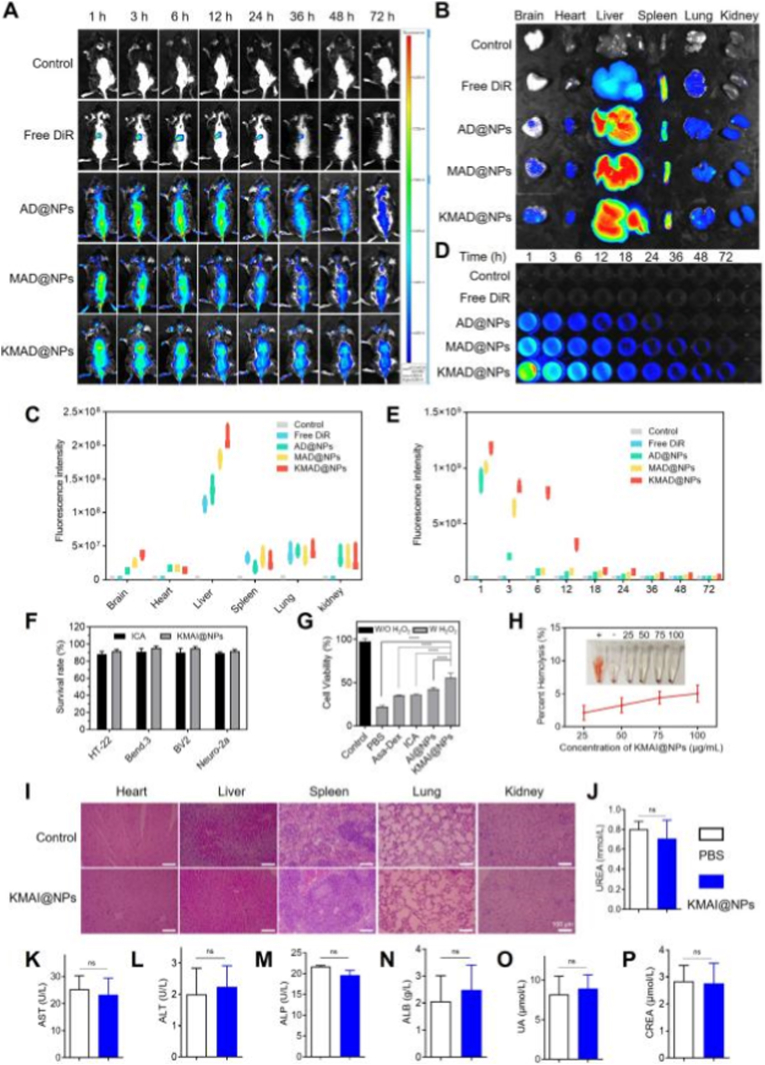
In vivo circulation behavior, brain accumulation, and biosafety evaluation of NPs. (**A**) Real-time fluorescence imaging of AD mice at predefined time points following intravenous injection of various formulations. (**B**) Ex vivo optical imaging of major organ tissues from AD mice at 72 h post-injection. (**C**) Semi-quantitative analysis of fluorescence signal intensity in ex vivo organ tissues from different treatment groups (n = 3). (**D**) Fluorescence signal analysis of a series of nanoparticles in rat blood over time. (**E**) Semi-quantitative analysis of fluorescence intensity from various formulations at predefined time points (n = 3). (**F**) Cytotoxic effects of different formulations on HT-22, Bend.3, BV.2, and Neuro-2a cells, as determined by cell viability assays (n = 6). (**G**) Cell viability in the presence of H_2_O_2_, showing the protective effect of the formulations (n = 6). (**H**) Photographs and quantitative analysis of hemolysis activity in red blood cells treated with KMAI@NPs at different concentrations (n = 3). (**I**) Representative HE staining images of heart, liver, spleen, lung, and kidney tissues from each mouse group (n = 3). Scale bar: 100 μm. (**J-P**) Biochemical analysis of serum parameters, including albumin (ALB), alkaline phosphatase (ALP), alanine aminotransferase (ALT), aspartate aminotransferase (AST), urea (UREA), creatinine (CREA), and uric acid (UA) levels (n = 3). Results are presented as mean ± SD (∗∗∗∗*P* < 0.0001). (For interpretation of the references to colour in this figure legend, the reader is referred to the Web version of this article.)

As shown in Fig. 4A, free DiR exhibited weak fluorescence signals that were primarily localized in the abdominal region and gradually diminished or disappeared within 36 h after injection, indicating rapid clearance and poor in vivo retention. In contrast, all nanoparticle formulations displayed prolonged fluorescence signals, with KMAD@NPs showing the most prominent and sustained accumulation in the brain region at all monitored time points. Notably, strong brain-associated fluorescence signals in the KMAD@NPs group persisted for up to 72 h, highlighting its superior brain-targeting and retention capability compared with other formulations.

Ex vivo fluorescence imaging of major organs harvested at 72 h post-injection further confirmed the in vivo imaging results (Fig. 4B). Semi-quantitative analysis of fluorescence intensity revealed significantly higher DiR accumulation in the brains of mice treated with KMAD@NPs compared with AD@NPs and MAD@NPs (Fig. 4C). These results indicate that dual-peptide modification markedly enhances nanoparticle accumulation in the brain.

To further evaluate the blood circulation behavior of the different formulations, blood samples were collected at predetermined time points following intravenous injection, and fluorescence intensity was measured to reflect circulating nanoparticle levels. As shown in Fig. 4D, fluorescence signals were detectable in the blood of all nanoparticle-treated groups over extended time periods, whereas no detectable signal was observed in the free DiR group at any time point. The absence of fluorescence in the free DiR group is attributed to its poor aqueous solubility and rapid recognition and clearance by the reticuloendothelial system (RES) [36]. In contrast, the hydrophilic PEG corona on the nanoparticle surface endows the formulations with a “stealth” effect, enabling evasion of rapid RES uptake and significantly prolonging systemic circulation [37]. Among the nanoparticle formulations, KMAD@NPs exhibited the strongest and most sustained fluorescence signals in circulation, likely owing to the presence of two PEG_2000_ segments in its architecture, which further enhance circulation stability. However, fluorescence signals from all formulations became negligible by 96 h post-injection, suggesting gradual clearance or redistribution to peripheral organs. Semi-quantitative analysis of fluorescence intensity over time is presented in Fig. 4E.

Collectively, these in vivo imaging results demonstrate that KMAD@NPs not only prolong systemic circulation but also exhibit markedly enhanced brain accumulation and BBB penetration, which are critical prerequisites for effective drug delivery in neurodegenerative disease therapy.

In addition to in vivo targeting performance, the biocompatibility and safety of KMAI@NPs were systematically evaluated, as favorable biosafety is essential for translational applications. The cytotoxicity of free ICA and KMAI@NPs was first assessed using a CCK-8 assay in HT-22, BV.2, Bend.3, and Neuro-2a cells. As shown in Fig. 4F, cell viability remained above 90% even at ICA concentrations up to 20 μM, indicating negligible cytotoxicity.

Furthermore, the protective effects of different formulations against oxidative stress were evaluated in BV.2 cells exposed to H_2_O_2_. H_2_O_2_ treatment induced severe cell death; however, pretreatment with Asa-Dex, ICA, AI@NPs, or KMAI@NPs significantly improved cell viability compared with the PBS-treated group (Fig. 4G), which can be attributed to their antioxidant properties. Notably, KMAI@NPs exhibited the most pronounced cytoprotective effect among all formulations (∗∗∗∗*P* < 0.0001).

Hemocompatibility was evaluated using a standard hemolysis assay. After incubation of red blood cells with KMAI@NPs at designated concentrations for 2 h, no visible damage to erythrocyte membranes was observed (Fig. 4H). The hemolysis ratios for all tested concentrations were below 5%, indicating excellent blood compatibility.

In vivo safety was further assessed by analyzing hematological parameters (Supplementary, Table S3) and serum biochemical markers, including liver and kidney function indicators (Fig. 4J–P). No significant differences were observed between KMAI@NPs-treated mice and control mice, suggesting the absence of apparent hematological, hepatic, or renal toxicity. Histological examination of major organs (heart, liver, spleen, lung, and kidney) from C57BL/6J mice treated with KMAI@NPs revealed no noticeable pathological abnormalities compared with control tissues (Fig. 4I), further supporting the favorable systemic safety profile of the nanoplatform.

Taken together, these results demonstrate that KMAI@NPs exhibit prolonged circulation, enhanced brain targeting, and excellent biosafety in vivo, underscoring their potential as a safe and effective nanotherapeutic platform for AD treatment.

### 2.4 Behavioral and cognitive improvements

**Fig. 5:**
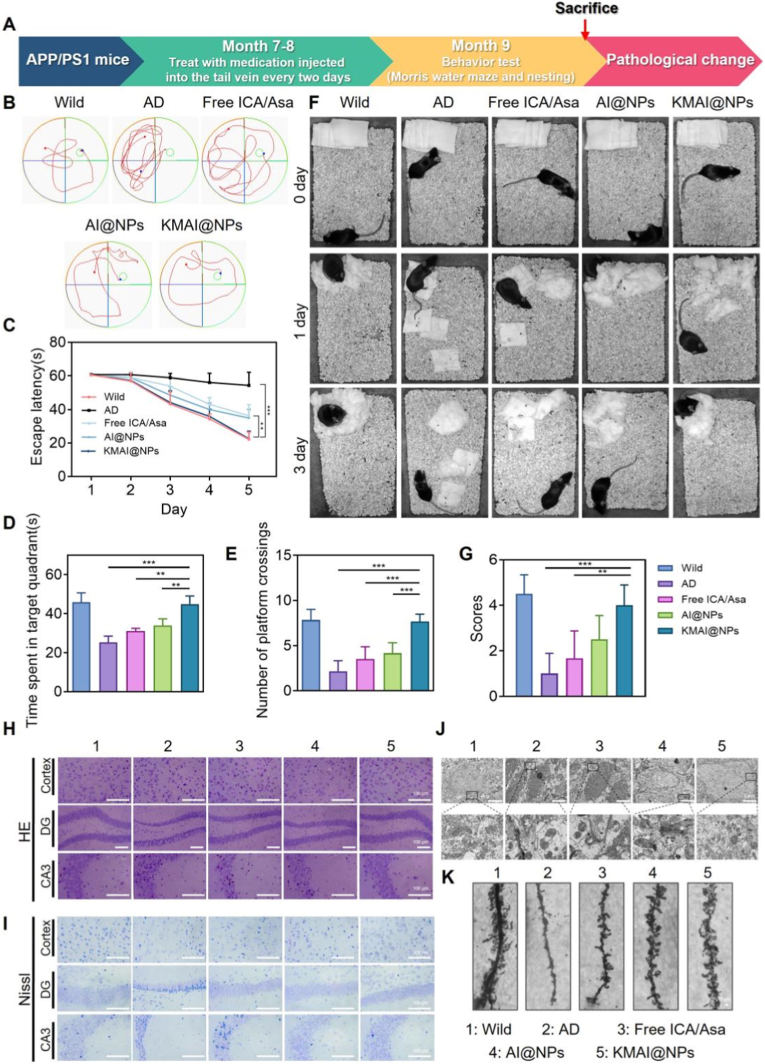
Timeline of drug treatment, pathological monitoring, and efficacy evaluation. (**A**) Experimental timeline for drug treatment, pathological monitoring, and efficacy assessment. (**B**) Representative swimming paths of mice in the Morris water maze. (**C**) Escape latency on day 5 of the Morris water maze test. (**D**) Time spent in the target quadrant during the Morris water maze test. (**E**) Number of platform crossings in the Morris water maze test. (**F**) Representative nesting images of mice from day 0 to day 3 in different treatment groups. (**G**) Analysis of nesting scores for the different treatment groups. (**H**) HE staining showing neuronal morphology in the hippocampus and cortex of mice treated with different formulations. Scale bar: 100 μm. (**I**) Nissl staining results of the hippocampus and cortex in mice treated with different formulations. Scale bar: 100 μm. (**J**) Representative transmission electron microscopy images of mitochondrial morphology in brain tissue sections. Scale bar: 5 μm. (**K**) Representative Golgi-Cox staining images of dendritic spines in the hippocampus from five groups. Scale bar: 1 μm. Results are reported as mean ± SD (n = 6, ∗∗*P* < 0.01, ∗∗∗*P* < 0.001).

As shown in Fig. 5A, we initiated treatment in APP/PS1 transgenic AD model mice at 7–8 months of age, administering KMAI@NPs via tail vein injection every other day for 8 weeks. C57BL/6J wild-type mice were used as normal controls. The therapeutic efficacy of KMAI@NPs was systematically compared to that of an mixture of free ICA and Asa, as well as to non-targeted AI@NPs. Spatial learning and memory were first evaluated by the Morris water maze. During the 5-day training period, APP/PS1 mice exhibited a markedly prolonged escape latency, indicating impaired learning ability. By contrast, AD mice treated with KMAI@NPs showed steadily shortening escape latencies over the training days, eventually approaching the performance of wild-type mice. This improvement was significantly greater than that observed in the free ICA/Asa mixture group or the non-targeted AI@NPs group (Fig. 5B and C).

In the spatial exploration test where the hidden platform was removed, APP/PS1 mice displayed a typical wall-hugging swimming pattern, reflecting severe impairment of spatial memory. In contrast, KMAI@NPs-treated mice spent significantly more time in the target quadrant, swam with more focused trajectories, and crossed the former platform location markedly more often (Fig. 5B–D, E). These indices nearly returned to wild-type levels and were substantially better than those of the free drug or non-targeted NPs groups.

Beyond the water maze, a nesting behavior test was used to further assess the mice's overall cognitive and functional status. Untreated APP/PS1 mice, due to cognitive decline, could barely construct a complete nest. In contrast, KMAI@NPs-treated AD mice showed a markedly restored nesting ability, with significantly higher nesting scores approaching those of the free drug or non-targeted NPs groups (Fig. 5F and G). Collectively, these behavioral results demonstrate that KMAI@NPs significantly alleviated cognitive deficits in APP/PS1 mice, with efficacy clearly superior to both the free drug combination and the non-targeted formulation.

### 2.5 Neuropathological and structural improvements

Cognitive improvement in AD is typically closely associated with the restoration of neuronal structure and function [38]. We therefore performed histological analyses of the brain tissues. Hematoxylin & eosin (HE) staining and Nissl staining revealed that, compared to wild-type mice, APP/PS1 model mice had significantly fewer neurons in the hippocampal dentate gyrus (DG) and CA3 regions, with a disorganized arrangement and obvious atrophy and necrosis. In the cortex, substantial neuronal damage and a reduction in Nissl bodies were also observed (Fig. 5H and I). After KMAI@NPs treatment, neuronal density and morphology in these regions showed marked improvement, Nissl body content rebounded, and tissue architecture became more orderly, indicating a significant rescue of neuronal loss and damage.

Mitochondria are a key site of ROS production and lipid peroxidation, and thus represent a “victim node” that is amplified during the vicious cycle of inflammation and iron dysregulation [[39], [40], [41]]. Transmission electron microscopy revealed severe mitochondrial damage in APP/PS1 mouse brains, such as fragmented cristae and vacuolization. Notably, KMAI@NPs treatment markedly alleviated these mitochondrial ultrastructural abnormalities (Fig. 5J).

Synaptic plasticity is closely related to memory formation. Golgi staining showed that APP/PS1 mice had a significantly lower dendritic spine density and impaired dendritic architecture, whereas KMAI@NPs treatment substantially improved dendritic morphology and increased spine density (Fig. 5K).

These findings indicate that the cognitive improvements conferred by KMAI@NPs were not limited to the behavioral level but were accompanied by the restoration of neuronal structural integrity, mitochondrial ultrastructure, and synaptic plasticity. This suggests that the therapeutic benefits of KMAI@NPs likely stem from systemic modulation of multiple pathological processes in the AD brain, rather than a single-target effect.

### 2.6 Reduction of Aβ pathology and ferroptosis markers

**Fig. 6:**
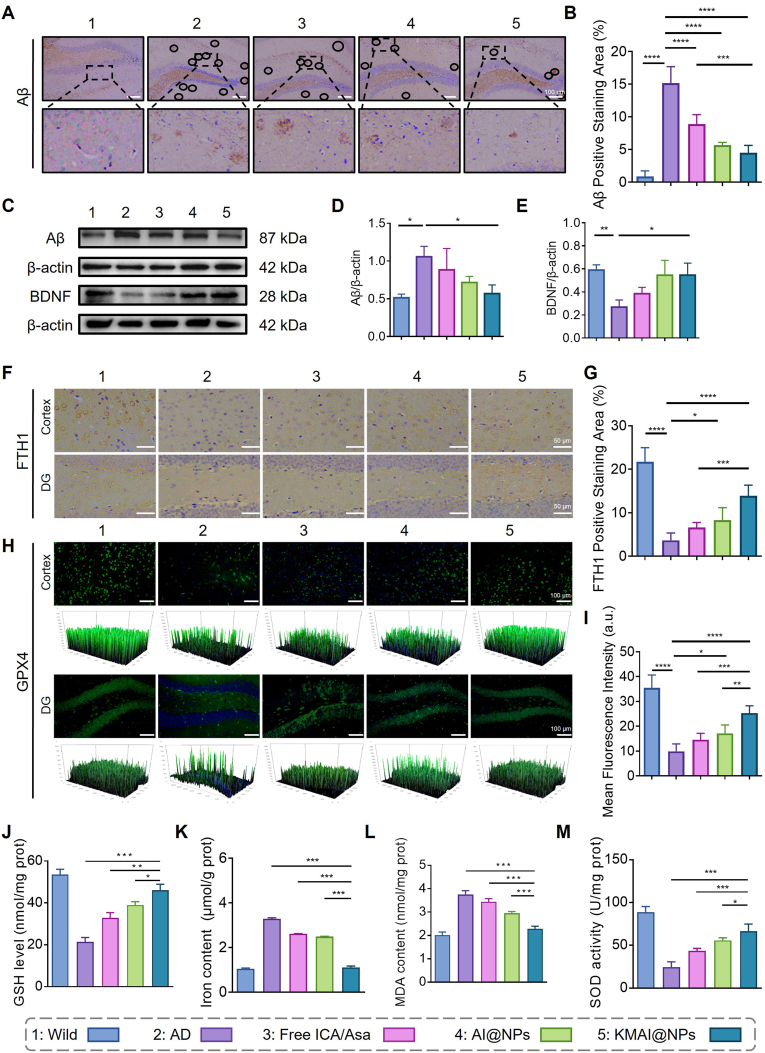
KMAI@NPs alleviate amyloid pathology and ferroptosis-related molecular abnormalities in APP/PS1 mice. (**A**) Representative immunohistochemical staining images of Aβ plaques in the hippocampus of wild-type mice, APP/PS1 mice, and APP/PS1 mice treated with free ICA/Asa, AI@NPs, or KMAI@NPs. Scale bar: 100 μm. (**B**) Quantitative analysis of Aβ-positive staining area. (**C**) Representative western blot images of Aβ and BDNF protein expression in brain tissues from different treatment groups. (**D**) Quantification of Aβ protein levels normalized to β-actin. (**E**) Quantification of BDNF protein expression. (**F**) Representative immunohistochemical staining images of FTH1 in the cortex and DG. Scale bar: 50 μm. (**G**) Quantitative analysis of FTH1-positive staining area. (**H**) Representative immunofluorescence images of GPX4 expression in the cortex and DG. Scale bar: 100 μm. (**I**) Quantitative analysis of GPX4-positive staining area and fluorescence intensity. (**J**) GSH levels in brain homogenates from different groups. (**K**) Total iron content in brain tissues. (**L**) MDA levels as an index of lipid peroxidation. (**M**) SOD activity in brain tissues. Results are presented as mean ± SD (n = 6, ∗*P* < 0.05, ∗∗*P* < 0.01, ∗∗∗*P* < 0.001, ∗∗∗∗*P* < 0.0001).

AD pathology is not driven by a single factor, but rather by inflammation, oxidative stress, and iron metabolic imbalance that are intertwined and self-amplifying [[42], [43], [44]]. We next examined whether KMAI@NPs treatment concurrently mitigated core pathological hallmarks of AD and ferroptosis-related molecular abnormalities. Immunohistochemistry and Western blot results showed that KMAI@NPs significantly reduced brain Aβ plaque deposition and amyloid-β protein levels (Fig. 6A–D), and the overall magnitude of Aβ reduction was greater than that achieved by free ICA/Asa or by AI@NPs. Meanwhile, brain-derived neurotrophic factor (BDNF) expression was upregulated in the KMAI@NPs group (Fig. 6C–E), suggesting an improved neuro-supportive microenvironment.

For indicators related to iron metabolism and lipid peroxidation, KMAI@NPs treatment significantly upregulated the expression of GPX4 and FTH1 (Fig. 6F–I), and increased the levels of key antioxidant molecules like glutathione (GSH) and superoxide dismutase (SOD) (Fig. 6J–M). Concurrently, malondialdehyde (MDA) levels were reduced (Fig. 6L), and total iron content in the brain were decreased (Fig. 6K). These results indicate that KMAI@NPs not only reduced the iron overload burden in the brain but also enhanced the clearance of lipid peroxides, thereby providing multi-pronged mitigation across interconnected processes involving iron overload, lipid peroxidation, and cellular damage.

### 2.7 Attenuation of neuroinflammation and glial activation

**Fig. 7:**
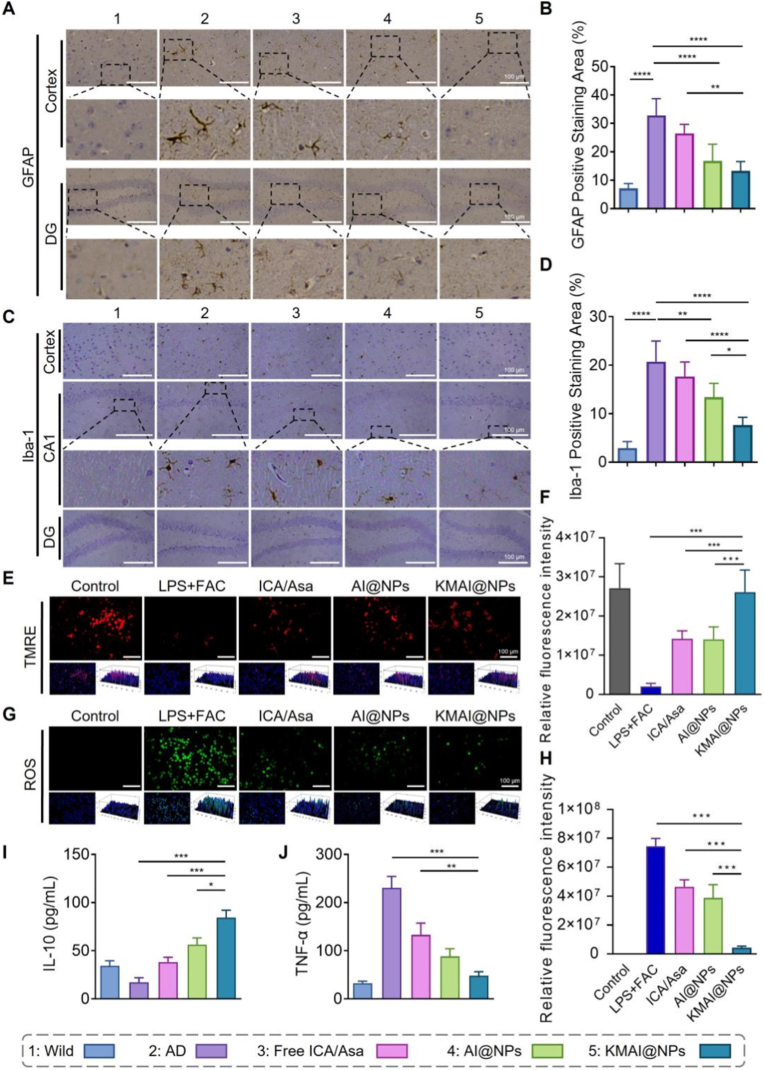
KMAI@NPs attenuate neuroinflammation and restore mitochondrial function in vivo and in vitro. (**A**) Representative immunohistochemical staining images of GFAP in the cortex and DG. Scale bar: 100 μm. (**B**) Quantitative analysis of GFAP-positive staining area (n = 6). (**C**) Representative immunohistochemical staining images of Iba-1 in the cortex and DG. Scale bar: 100 μm. (**D**) Quantitative analysis of Iba-1-positive staining area (n = 6). (**E**) Representative TMRE staining images of BV.2 microglial cells. Scale bar: 100 μm. (**F**) Quantitative analysis of TMRE fluorescence intensity (n = 3). (**G**) Representative intracellular ROS staining images of BV.2 cells under LPS + FAC stimulation following different treatments. Scale bar: 100 μm. (**H**) Quantitative analysis of intracellular ROS levels (n = 3). (**I**) TNF-α levels in brain tissues from different treatment groups (n = 6). (**J**) IL-10 levels in brain tissues from different treatment groups (n = 6). Results are reported as mean ± SD (∗*P* < 0.05, ∗∗*P* < 0.01, ∗∗∗*P* < 0.001, ∗∗∗∗*P* < 0.0001).

We then evaluated the effect of KMAI@NPs treatment on the neuroinflammatory microenvironment. Immunohistochemical staining was used to assess glial cell activation in the brains of AD mice. Glial fibrillary acidic protein (GFAP) and Iba-1 were examined as canonical markers for activated astrocytes and microglia, respectively. As shown in Fig. 7A–D, APP/PS1 model mice exhibited markedly increased GFAP- and Iba-1-positive areas in the cortex and DG, indicating abnormal, excessive activation of astrocytes and microglia. In contrast, after KMAI@NPs treatment, GFAP and Iba-1 positive areas were greatly reduced, with the reduction far more pronounced than in the free ICA/Asa and AI@NPs groups, approaching levels seen in wild-type mice. This demonstrates that KMAI@NPs effectively alleviated the abnormal glial hyperactivation in the AD brain, thereby reducing neuroinflammatory responses.

In addition, KMAI@NPs treatment significantly decreased the brain expression of the pro-inflammatory cytokine TNF-α while increasing the level of the anti-inflammatory cytokine IL-10 (Fig. 7I and J). This shift from a proinflammatory-dominant to an anti-inflammatory-favored profile suggests that KMAI@NPs restrained excessive microglial activation and reduced inflammatory mediator production, thereby alleviating chronic inflammation and oxidative stress-related neuronal stress [45].

To elucidate the cellular basis of the above in vivo anti-inflammatory effects, we established an LPS + FAC-induced BV.2 microglial inflammation model to simulate an AD-related pro-inflammatory, iron-overloaded microenvironment. Mitochondrial function and oxidative stress in these cells were then assessed. Tetramethylrhodamine ethyl ester (TMRE) staining revealed that LPS + FAC exposure markedly diminished the mitochondrial membrane potential in BV.2 cells, as indicated by a loss of TMRE fluorescence (Fig. 7E and F). This implies severe mitochondrial dysfunction under inflammatory stress. Notably, treatment with KMAI@NPs substantially restored the TMRE fluorescence signal in BV.2 cells, indicating an improvement in mitochondrial function; the recovery was much more pronounced than that observed with free ICA/Asa or with AI@NPs. In parallel, staining with a ROS-sensitive probe showed that intracellular ROS levels were dramatically elevated by LPS + FAC (Fig. 7G and H). KMAI@NPs treatment significantly reduced ROS accumulation in BV.2 cells, with a greater reduction than achieved by the other treatments. These data suggest that KMAI@NPs not only suppress inflammation but actively rescue microglial cells from inflammatory mitochondrial dysfunction and oxidative stress, shifting them from a persistently pro-inflammatory state back toward a more homeostatic, repair-oriented functional status.

### 2.8 Microglial phenotype reprogramming

**Fig. 8:**
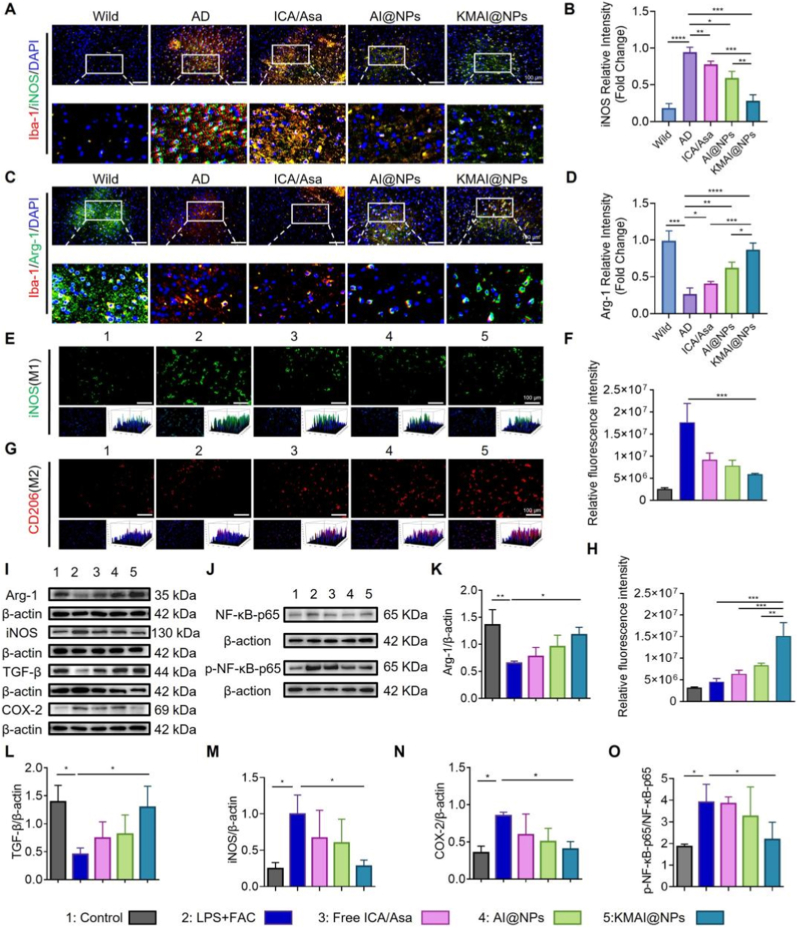
KMAI@NPs reprogram microglial phenotypes toward an anti-inflammatory M2 state in vivo and in vitro. (**A**) Representative immunofluorescence images of iNOS (M1 marker) and Iba-1 co-staining in the cortex. Scale bar: 100 μm. (**B**) Semi-quantitative analysis of iNOS^+^ (n = 6). (**C**) Representative immunofluorescence images of Arg-1 (M2 marker) and Iba-1 co-staining in the cortex of different treatment groups. Scale bar: 100 μm. (**D**) Semi-quantitative analysis of Arg-1^+^ (n = 6). (**E**) Representative immunofluorescence images of BV.2 microglial cells showing iNOS expression after LPS + FAC stimulation and different treatments. Scale bar: 100 μm. (**F**) Semi-quantitative analysis of iNOS fluorescence intensity in BV.2 cells. (**G**) Representative immunofluorescence images of BV.2 cells showing CD206 (M2 marker) expression under the same conditions (n = 3). Scale bar: 100 μm. (**H**) Semi-quantitative analysis of CD206 fluorescence intensity in BV.2 cells (n = 3). (**I–J**) Representative Western blot images of proteins (Arg-1, iNOS, TGF-β, COX-2) and NF-κB p65/phospho-p65 in BV.2 cells under LPS + FAC stimulation following different treatments. (**K–N**) Quantitative analysis of protein expression levels corresponding to panel (I) (n = 3). (**O**) Quantification of the p-NF-κB p65/NF-κB p65 ratio (n = 3). Results are reported as mean ± SD (∗*P* < 0.05, ∗∗*P* < 0.01, ∗∗∗*P* < 0.001, ∗∗∗∗*P* < 0.0001).

Having established that KMAI@NPs markedly improve the overall pathological phenotype and cognition in APP/PS1 mice, we next focused on their impact on microglia, the central hub of the AD pathological network. Aberrant microglial polarization is thought to be a key driver of the vicious cycle linking inflammation, oxidative stress, and iron dysregulation [[46], [47], [48]]. We therefore first evaluated how KMAI@NPs treatment influenced microglial phenotype in vivo. Immunofluorescence co-staining for the microglial marker Iba-1 together with M1/M2 polarization markers revealed pronounced pro-inflammatory activation of microglia in AD model mice. There was a dramatic increase in iNOS^+^/Iba-1^+^ cells (classically activated M1 microglia) accompanied by a significant decrease in Arg-1^+^/Iba-1^+^ cells (alternatively activated M2 microglia) compared to wild-type mice (Fig. 8A–D).

Strikingly, after KMAI@NPs treatment, the microglial phenotype distribution was substantially reshaped. On one hand, the number of iNOS^+^/Iba-1^+^ M1 microglia was greatly reduced; on the other hand, the proportion of Arg-1^+^/Iba-1^+^ M2 microglia rose significantly, approaching, or even partially restoring to, the levels seen in wild-type mice. Quantitative analysis confirmed this shift (Fig. 8B–D). Notably, KMAI@NPs produced a much stronger effect on microglial repolarization than either free ICA/Asa or non-targeted AI@NPs, highlighting that efficient brain-targeted delivery is crucial for effective immune reprogramming.

To further validate the direct effect of KMAI@NPs on microglial polarization and its functional consequences, we utilized the LPS + FAC-induced BV.2 cell model of inflammatory polarization. Fluorescence imaging showed that LPS + FAC strongly drove BV.2 cells toward a pro-inflammatory M1 phenotype, evidenced by markedly enhanced iNOS signals and a concurrent reduction in the M2 marker CD206 (Fig. 8E–G). By contrast, KMAI@NPs treatment significantly suppressed iNOS expression while markedly increasing CD206 signals (Fig. 8F–H). This indicates that under sustained inflammatory stimulation, KMAI@NPs effectively redirect microglia from a pro-inflammatory M1 state toward an anti-inflammatory, reparative M2 state. Importantly, this phenotype shift was most pronounced with KMAI@NPs, underscoring the importance of targeted delivery and multi-pathway coordination in achieving microglial reprogramming.

We further examined changes in microglial inflammatory signaling networks. LPS + FAC treatment markedly upregulated inflammatory proteins such as iNOS and COX-2, accompanied by a significant increase in NF-κB p65 phosphorylation, indicating that the classical NF-κB-mediated inflammatory amplification pathway was strongly activated (Fig. 8I, J, M − O). In contrast, KMAI@NPs treatment significantly suppressed the expression of these pro-inflammatory mediators and greatly reduced the p-NF-κB p65/total p65 ratio (Fig. 8O), while simultaneously increasing the levels of anti-inflammatory/resolution markers like Arg-1 and TGF-β (Fig. 8K and L). These findings suggest that KMAI@NPs effectively blocked the NF-κB-driven inflammatory positive feedback loop, thereby stabilizing microglia in an anti-inflammatory phenotype at the molecular level.

We also analyzed the secretory profile of BV.2 microglia (Supplementary, Fig. S5). LPS + FAC stimulation caused massive release of pro-inflammatory cytokines (TNF-α, IL-1β, IL-6) and a significant increase in nitric oxide (NO), while suppressing the secretion of the anti-inflammatory cytokine IL-10 (Supplementary, Fig. S5A–E). In contrast, KMAI@NPs treatment not only significantly lowered the levels of TNF-α, IL-1β, IL-6 and NO, but also markedly elevated IL-10 secretion. The extent of these changes was much greater than that observed with free ICA/Asa or with AI@NPs. This demonstrates that KMAI@NPs effectively suppress NF-κB-mediated inflammatory amplification, thereby driving microglia toward an anti-inflammatory, homeostasis-maintaining functional state.

Together, these results indicate that KMAI@NPs do not act merely by passively dampening inflammatory signals; rather, they actively reshape the functional phenotype of microglia. Through microglia-centered immune reprogramming, KMAI@NPs modulated the tightly coupled pathological network involving inflammation, metabolism, and oxidative stress in AD. Such microglia-centric reprogramming not only helps halt the sustained amplification of neuroinflammation, but may also create a more favorable environment for restoring iron homeostasis and rebuilding antioxidant defenses.

### 2.9 Nrf2 pathway activation and ferroptosis suppression

**Fig. 9:**
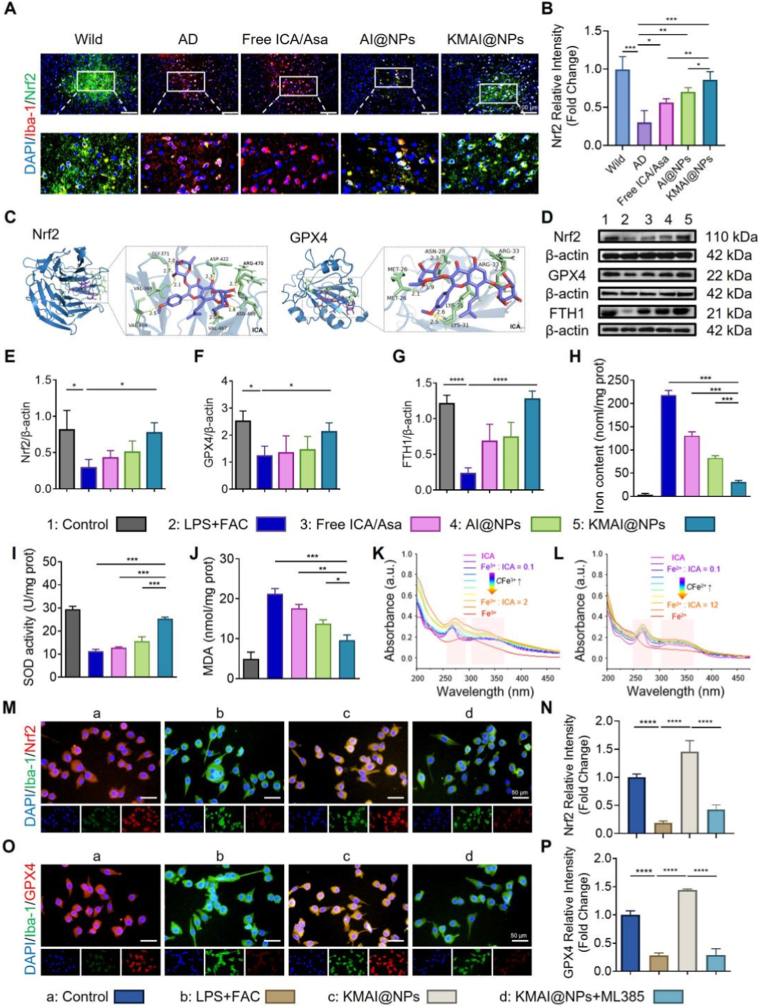
KMAI@NPs activate the Nrf2/GPX4 axis to restore iron homeostasis and suppress ferroptosis in microglia. (**A**) Representative immunofluorescence images of Nrf2 and Iba-1 co-staining in the cortex. Scale bar: 100 μm. (**B**) Semi-quantitative analysis of Nrf2^+^ in different treatment groups (n = 6). (**C**) Molecular docking models illustrating the predicted binding modes of ICA with Nrf2 and GPX4. (**D**) Representative western blot images of Nrf2, GPX4, and FTH1 expression in BV.2 cells. (**E–G**) Quantitative analysis of Nrf2, GPX4, and FTH1 protein expression levels corresponding to panel (D) (n = 3). (**H**) Intracellular iron content in BV.2 cells under different treatment conditions (n = 3). (**I**) SOD activity in BV.2 cells (n = 3). (**J**) MDA levels in BV.2 cells (n = 3). (**K–L**) UV-vis absorbance spectra of the titration of ICA with (K) Fe^2+^ and (L) Fe^3+^. (**M**) Representative immunofluorescence images of Nrf2 expression in BV.2 microglial cells. Scale bar: 50 μm. (**N**) Semi-quantitative analysis of Nrf2 fluorescence intensity (M) (n = 3). (**O**) Representative immunofluorescence images of GPX4 expression in BV.2 cells. Scale bar: 50 μm. (**P**) Semi-quantitative analysis of GPX4 fluorescence intensity corresponding to panel (O) (n = 3). Results are reported as mean ± SD (∗*P* < 0.05, ∗∗*P* < 0.01, ∗∗∗*P* < 0.001, ∗∗∗∗*P* < 0.0001).

In the previous sections, we demonstrated that KMAI@NPs significantly improved the pathological features and cognitive deficits in APP/PS1 mice, while effectively reprogramming the inflammatory phenotype of microglia. Because microglial activation in AD is closely associated with oxidative stress and iron dysregulation, we next examined whether KMAI@NPs could alleviate ferroptosis-related injury in microglia. This is particularly relevant because microglia act as both regulators and vulnerable targets within the inflammatory and iron-related pathological network [[13], [14], [15], [16]]. Given that Nrf2 is a key transcription factor involved in the maintenance of redox homeostasis and iron metabolic balance, we next investigated whether KMAI@NPs could protect microglia against ferroptosis through Nrf2-related signaling [[49], [50], [51]]. Immunofluorescence analysis revealed that in AD model mice, the proportion of Nrf2^+^ microglia was significantly lower than in wild-type mice, indicating that microglia's endogenous antioxidant defense (*via* Nrf2) is suppressed in the AD brain microenvironment (Fig. 9A).

Notably, after KMAI@NPs treatment, Nrf2 expression in microglia was markedly restored, to levels well above those seen in the free ICA/Asa and non-targeted AI@NPs groups (Fig. 9A and B). This demonstrates that targeted nanoparticle delivery can effectively restore impaired Nrf2 signaling in AD microglia in vivo, which is associated with improved antioxidant capacity and iron-homeostasis regulation.

To further delineate this effect at the cellular and molecular level, we examined the direct regulation of the Nrf2 pathway by KMAI@NPs in BV.2 microglia. Under LPS + FAC-induced inflammation, iron overload conditions, Western blots showed that LPS + FAC treatment dramatically downregulated Nrf2 protein and its downstream ferroptosis-defense proteins GPX4 and FTH1 in BV.2 cells. KMAI@NPs treatment, however, significantly restored the levels of Nrf2, GPX4, and FTH1, with much stronger effects than those observed with the free drug or non-targeted NPs (Fig. 9D–G). Consistent with these changes, LPS + FAC triggered a significant accumulation of intracellular iron, whereas KMAI@NPs treatment markedly reduced the iron content in BV.2 cells, outperforming the free drug and non-targeted NPs in reducing iron overload (Fig. 9H). These results indicate that KMAI@NPs directly activate the Nrf2/GPX4/FTH1 axis in microglia, thereby restoring the cells’ iron-buffering capacity and relieving iron overload.

Restoration of iron homeostasis would be expected to coincide with a reduction in oxidative stress. Indeed, in BV.2 cells KMAI@NPs treatment significantly increased the activity of the antioxidant enzyme SOD while concurrently reducing the level of MDA, a lipid peroxidation end-product (Fig. 9I and J). These findings suggest that KMAI@NPs, by simultaneously modulating iron metabolism and the antioxidant system, effectively suppress iron-dependent lipid peroxidation, thereby lowering the risk of ferroptotic cell death.

Since ICA, one of the therapeutic cargos in KMAI@NPs, possesses a flavonoid structure, we hypothesized that it might directly chelate iron and thereby contribute to regulation of iron homeostasis. UV-Vis spectroscopy was performed to test this possibility. We observed that as the concentration of Fe^2+^ or Fe^3+^ increased, ICA's characteristic absorption peak at 268 nm gradually diminished, and a distinct ligand-to-metal charge transfer (LMCT) band emerged in the 300–350 nm range (Fig. 9K and L) [[52], [53], [54]]. This indicates that ICA can form stable complexes with iron ions in both ferrous and ferric states. This result chemically confirms ICA's ability to directly alleviate iron overload, providing a plausible explanation for its observed effect in reducing iron accumulation in cells (Supplementary, Fig. S6).

To verify the causal role of Nrf2 in KMAI@NPs’ anti-ferroptotic effects, we employed the Nrf2-specific inhibitor ML385 in the BV.2 model. Immunofluorescence showed that KMAI@NPs treatment strongly enhanced the expression of Nrf2 and GPX4 in BV.2 cells, but this upregulation was markedly blunted by co-treatment with ML385 (Fig. 9M − P). Quantitative analysis confirmed that inhibiting Nrf2 significantly diminished the protective effect of KMAI@NPs on GPX4, indicating that the antioxidant and anti-ferroptosis benefits of KMAI@NPs are largely dependent on Nrf2 activation.

Furthermore, molecular docking simulations were performed to explore potential interactions between ICA and key ferroptosis-regulating proteins. The results revealed that ICA has good binding affinity for both Nrf2 and GPX4, forming multiple hydrogen bonds with critical amino acid residues. This provides a structural rationale for ICA-mediated regulation of the Nrf2/GPX4 signaling axis (Fig. 9C, Supplementary, Table S4).

Together, these findings indicate that KMAI@NPs restore Nrf2/GPX4 signaling in microglia, accompanied by reduced iron accumulation, improved redox balance, and suppression of ferroptosis. Combined with the previously observed reprogramming of inflammatory phenotypes, these results suggest that KMAI@NPs exert coordinated regulatory effects on microglial state and survival under AD-related pathological conditions. This provides a mechanistic basis for the observed neuroprotective effects and also supports further investigation into whether such protection extends to specific microglial subpopulations.

### 2.10 Sustaining M2 microglia via ferroptosis inhibition

The above results showed that KMAI@NPs not only reprogram microglia from a pro-inflammatory M1 state toward an anti-inflammatory/repair-oriented M2 state, but also protect microglia against ferroptosis through activation of the Nrf2/GPX4 pathway. However, emerging evidence suggests that M2-polarized microglia are paradoxically more susceptible to ferroptotic injury under iron-rich and highly oxidative conditions, which may limit the persistence of their neuroprotective functions [[55], [56], [57]]. Therefore, after establishing the overall protective effect of KMAI@NPs on microglia, we further investigated whether this protection could extend to M2-polarized microglia, thereby preserving the functions of this beneficial subpopulation.

**Fig. 10:**
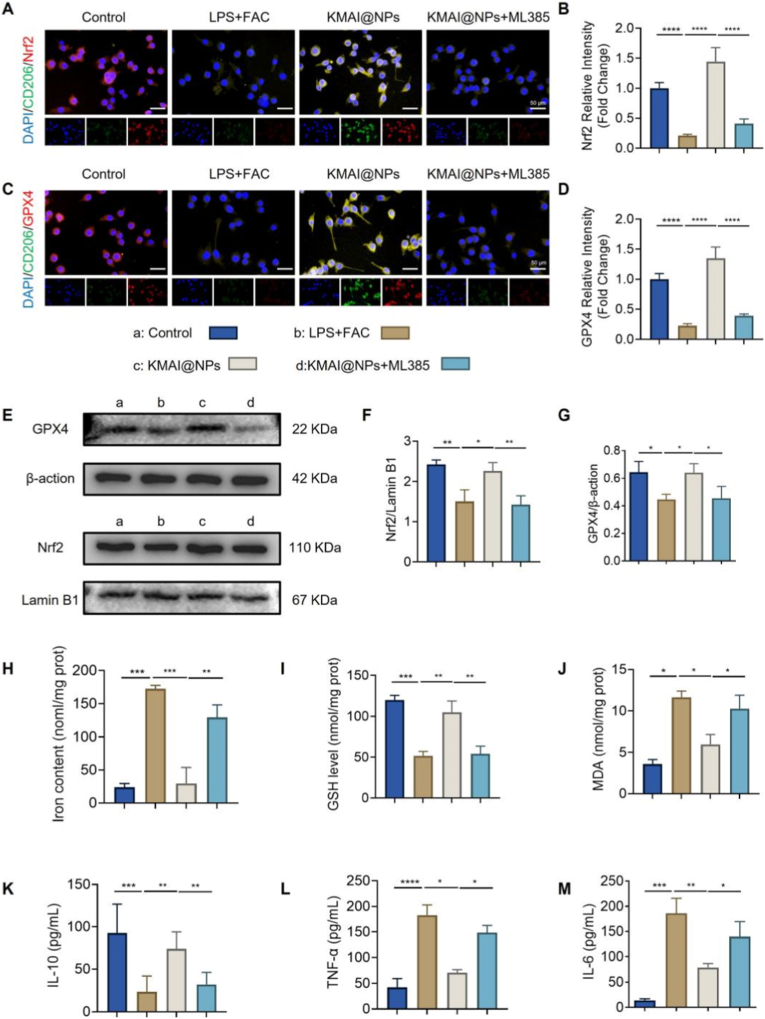
KMAI@NPs suppress ferroptosis vulnerability in M2-polarized microglia via Nrf2-dependent mechanisms. (**A**) Representative immunofluorescence images showing Nrf2 expression in CD206^+^ M2-polarized BV.2 microglial cells. Scale bar: 50 μm. (**B**) Semi-quantitative analysis of Nrf2 fluorescence intensity corresponding to panel (A). (**C**) Representative immunofluorescence images of GPX4 expression in CD206^+^ M2-polarized BV.2 cells. Scale bar: 50 μm. (**D**) Semi-quantitative analysis of GPX4 fluorescence intensity corresponding to panel (C). (**E**) Representative western blot images showing cytoplasmic and nuclear Nrf2 expression, as well as GPX4 protein levels. (**F**) Quantitative analysis of nuclear Nrf2 levels normalized to Lamin B1. (**G**) Quantitative analysis of GPX4 protein expression levels corresponding to panel (E). (**H**) Intracellular iron content in M2-polarized BV.2 cells under different treatment conditions. (**I**) Intracellular GSH levels. (**J**) MDA levels. (**K–M**) Levels of secreted cytokines IL-10, TNF-α, and IL-6. Results are reported as mean ± SD (n = 3, ∗*P* < 0.05, ∗∗*P* < 0.01, ∗∗∗*P* < 0.001, ∗∗∗∗*P* < 0.0001).

In the LPS + FAC BV.2 model, immunofluorescence showed that KMAI@NPs treatment greatly enhanced Nrf2 expression and nuclear translocation specifically in CD206^+^ (M2) microglia, whereas this effect was significantly weakened by the addition of ML385 (Fig. 10A and B). Similarly, KMAI@NPs markedly upregulated GPX4 in M2 microglia, and this upsurge was largely reversed by ML385 co-treatment (Fig. 10C and D). These results suggest that under inflammatory and iron-overload stress, KMAI@NPs rely on Nrf2 activation to confer ferroptosis resistance to M2 microglia. Consistent with the immunofluorescence findings, Western blot analysis confirmed that under LPS + FAC conditions, KMAI@NPs treatment promoted Nrf2 nuclear translocation (increased Nrf2/Lamin B1 ratio) and restored GPX4 protein expression; when Nrf2 was inhibited by ML385, these protective effects were almost completely abrogated (Fig. 10E–G). Thus, KMAI@NPs effectively activate the endogenous antioxidant and anti-ferroptotic defense programs in M2 microglia even in the face of inflammatory iron overload, and this activation is critically dependent on Nrf2.

Functionally, LPS + FAC treatment triggered classic ferroptotic changes in BV.2 cells, including significant iron accumulation, GSH depletion, and elevated lipid peroxidation, as reflected by increased malondialdehyde (MDA) levels, indicating robust activation of the ferroptosis pathway (Fig. 10H–J). KMAI@NPs treatment counteracted these changes by markedly reducing intracellular iron accumulation, restoring GSH levels, and suppressing MDA production. When Nrf2 was inhibited by ML385, these protective effects of KMAI@NPs were notably attenuated (Fig. 10H–J), further indicating that Nrf2 is a pivotal mediator of KMAI@NPs’ ability to inhibit ferroptosis in microglia. Moreover, KMAI@NPs treatment preserved the anti-inflammatory function of microglia by significantly increasing the secretion of the anti-inflammatory cytokine IL-10 while decreasing the release of the pro-inflammatory cytokines TNF-α and IL-6. With Nrf2 blocked, however, this advantageous cytokine profile was substantially dampened (Fig. 10K–M). This indicates that KMAI@NPs not only drive microglia toward an M2 phenotype, but by preventing ferroptosis they ensure that these M2 cells maintain a stable anti-inflammatory and immunoregulatory function.

In summary, KMAI@NPs exert a dual regulatory mechanism in microglia. On one hand, they promote microglial polarization from a pro-inflammatory M1 state to an anti-inflammatory/repair-oriented M2 state; On the other hand, they activate the Nrf2/GPX4 pathway in M2 microglia under inflammatory and iron-overload conditions, thereby reducing ferroptosis susceptibility and preserving neuroprotective functions. This finding offers a mechanistic perspective on the interconnected processes involving inflammation, iron imbalance, and microglial functional impairment in AD, and further highlights the therapeutic potential of KMAI@NPs through coordinated regulation of microglial metabolic and inflammatory activities. Integrating both in vivo and in vitro results, we propose a microglia-centered cascade model to illustrate the multi-level actions of KMAI@NPs within the intertwined pathological features of AD.

Under AD-related inflammatory and iron-overload conditions, microglia are initially driven into a classically activated M1 state characterized by NF-κB activation and elevated release of pro-inflammatory factors (Fig. 8). This pro-inflammatory phenotype not only amplifies local neuroinflammation, but also induces increased iron uptake and accumulation, exacerbates lipid peroxidation, and leads to mitochondrial dysfunction. Together, these changes push microglia into a ferroptosis-prone state. Such mutual reinforcement between inflammation and iron dysmetabolism constitutes a key pathological basis that continuously drives neuronal damage during AD progression.

The introduction of KMAI@NPs triggers a directional reversal of this pathological cascade. First, through targeted delivery and multi-pathway synergy, KMAI@NPs significantly suppress the NF-κB-mediated pro-inflammatory signaling axis, downregulating iNOS, COX-2, TNF-α and other inflammatory mediators, while simultaneously shifting microglial polarization from M1 to M2 (Fig. 8). However, M2 polarization alone is not sufficient to ensure sustained neuroprotection. In environments characterized by ongoing inflammation and iron overload, M2 microglia are paradoxically more susceptible to ferroptosis, leading to a rapid loss of their protective functions.

At this critical juncture, KMAI@NPs activate the Nrf2/GPX4 anti-ferroptosis axis, achieving deeper control over microglial fate (Fig. 9, Fig. 10). KMAI@NPs markedly promoted Nrf2 expression and nuclear translocation in microglia, upregulated key proteins such as GPX4 and FTH1 involved in maintaining iron homeostasis and defending against lipid peroxidation, and concomitantly reduced intracellular free iron accumulation, restored GSH levels, and inhibited MDA production, thereby effectively blocking the ferroptosis pathway. Nrf2 inhibition experiments confirmed that this protective effect is necessary for KMAI@NPs to maintain M2 microglial functional stability.

Therefore, KMAI@NPs do not act simply through “anti-inflammation” or “pro-M2 polarization” alone, but by reprogramming the core amplifying nodes of the microglial inflammation–iron metabolism network, they simultaneously achieve phenotype remodeling and cell-fate protection. This multi-tiered cascade regulation ultimately translated into tangible therapeutic outcomes in AD mice, as evidenced by the restoration of neuronal structure, recovery of synaptic plasticity, and significant improvement in cognitive function (Fig. 5, Fig. 6, Fig. 7). In effect, KMAI@NPs severed the pathological positive feedback loop driven by inflammation and iron dysregulation at the systems level. This cascade model suggests that, in a complex neurodegenerative disease such as AD, targeting microglial metabolic and fate-regulating processes, rather than individual inflammatory factors or single-pathway interventions, may provide more sustained and comprehensive therapeutic benefits.

Although the present study demonstrated that KMAI@NPs exert neuroprotective effects by regulating microglial inflammatory phenotypes and inhibiting ferroptosis, the underlying molecular regulatory network still requires further clarification. In particular, systematic identification of key upstream and downstream regulatory genes of the Nrf2/GPX4 pathway will help deepen the understanding of ferroptosis regulation in microglia. In future studies, our group will combine transcriptomic sequencing, ChIP-seq, and related approaches to systematically identify key molecules that act in coordination with this pathway, thereby providing additional potential targets for precise intervention in AD. In addition, the molecular relationship between microglial polarization and ferroptosis inhibition remains to be further elucidated. Our group will also use experimental strategies such as conditioned medium to further investigate the functional connection and regulatory network between these two processes.

## 3 Conclusions

Addressing the self-amplifying pathological network characterized by the tight coupling of inflammation, oxidative stress, and iron dyshomeostasis in AD, this study developed a microglia-centered nanotherapeutic strategy to enable coordinated intervention of this complex system. The engineered KMAI@NPs exhibited favorable circulation stability, markedly enhanced brain accumulation, and reliable biocompatibility and systemic safety in vivo. In APP/PS1 transgenic AD mice, repeated administration of KMAI@NPs significantly improved spatial learning, memory, and nesting behavior, accompanied by robust neuroprotection at the histological and ultrastructural levels, including alleviation of neuronal damage in the hippocampus and cortex, restoration of mitochondrial ultrastructure, and recovery of synapse-associated structural features.

At the pathological level, KMAI@NPs concurrently mitigated multiple hallmarks of AD, including reduced Aβ deposition, increased BDNF expression, and coordinated improvement of iron metabolism and lipid peroxidation abnormalities, as evidenced by decreased brain iron content, upregulated GPX4 and FTH1, enhanced GSH and SOD levels, and reduced MDA accumulation. These findings indicate that KMAI@NPs exert synergistic regulation of oxidative stress and iron homeostasis.

Mechanistic investigations further revealed that KMAI@NPs markedly reshaped microglial functional states in vivo by suppressing pro-inflammatory M1 features and promoting enrichment of anti-inflammatory/repair-associated M2 phenotypes. In LPS- and iron-overload–challenged BV.2 microglia, KMAI@NPs effectively attenuated NF-κB-mediated inflammatory amplification, reduced ROS generation, restored mitochondrial function, and activated the Nrf2/GPX4 axis, thereby alleviating intracellular iron accumulation and inhibiting lipid peroxidation. Pharmacological inhibition of Nrf2 further confirmed that the protective effects of KMAI@NPs against iron overload and oxidative damage are highly dependent on Nrf2 pathway activation.

Notably, given the increased susceptibility of anti-inflammatory microglia to ferroptosis under iron-rich and oxidative conditions, this study demonstrates that KMAI@NPs significantly reduce ferroptosis vulnerability in CD206^+^ microglia in an Nrf2-dependent manner, thereby maintaining their anti-inflammatory secretory and immunomodulatory functions. These results indicate that KMAI@NPs not only reprogram microglial functional states but also enhance their functional stability by regulating ferroptosis-associated susceptibility.

In summary, by targeting microglia as a key regulatory node within the AD pathological network, KMAI@NPs achieve coordinated modulation of inflammatory phenotypes and iron/redox homeostasis, effectively weakening the mutually reinforcing interplay between inflammation and iron dysregulation and ultimately translating into neuroprotection and cognitive improvement. This work provides a grounded framework for the design of multi-target nanotherapeutic strategies in neurodegenerative diseases.

## 4 Materials and methods

Materials and methods are described in the Supplementary.

## CRediT authorship contribution statement

**Yang Yu:** Funding acquisition, Writing – original draft, Writing – review & editing. **Jun-jie Yu:** Methodology, Writing – review & editing. **Shuai-wen Ding:** Investigation. **Ge Zhang:** Visualization. **Yang Liu:** Funding acquisition, Supervision. **Rui-bo Guo:** Formal analysis. **Juan Zang:** Conceptualization. **Xiang-xuan Zhao:** Writing – original draft, Writing – review & editing. **Xue-tao Li:** Funding acquisition, Writing – review & editing. **Liang Kong:** Methodology, Writing – original draft, Writing – review & editing.

## Declaration of competing interest

The authors declare that they have no known competing financial interests or personal relationships that could have appeared to influence the work reported in this paper.

## Data Availability

No data was used for the research described in the article.
