# Supplementary material for: An engineered ROS-responsive cascade nanoplatform delays Alzheimer's disease progression via Nrf2/GPX4-mediated microglial functional reprogramming
Source: Mater Today Bio. 2026 Mar 27;38:103055. doi: 10.1016/j.mtbio.2026.103055 (PMC13067124; doi:10.1016/j.mtbio.2026.103055)
Supplement: Multimedia component 1 [file mmc1.docx]

**Supplementary**


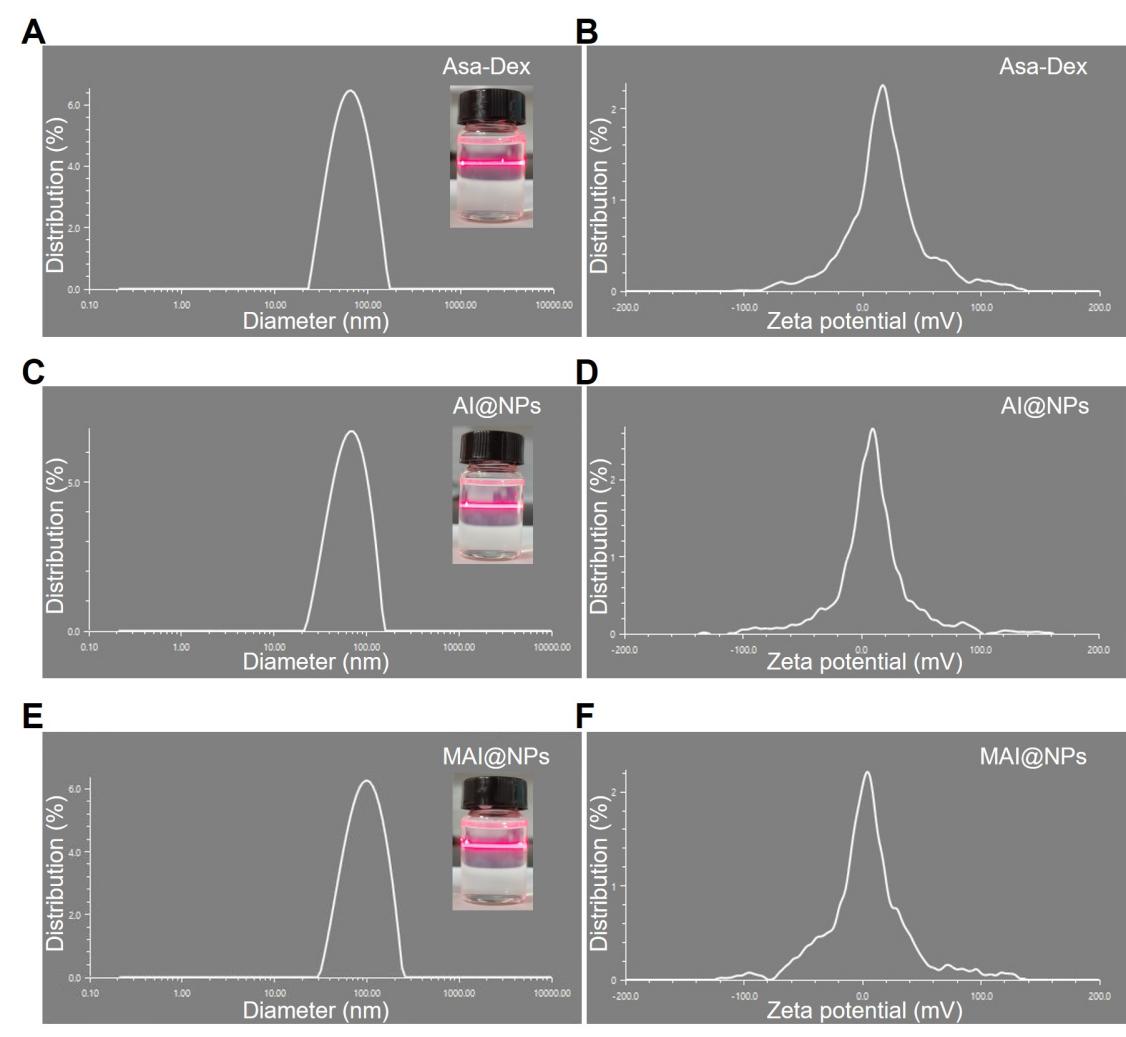


**Fig. S1.** (**A, C, E**) Representative images of particle sizes. (**B, D, F**) Representative images of zeta potential.


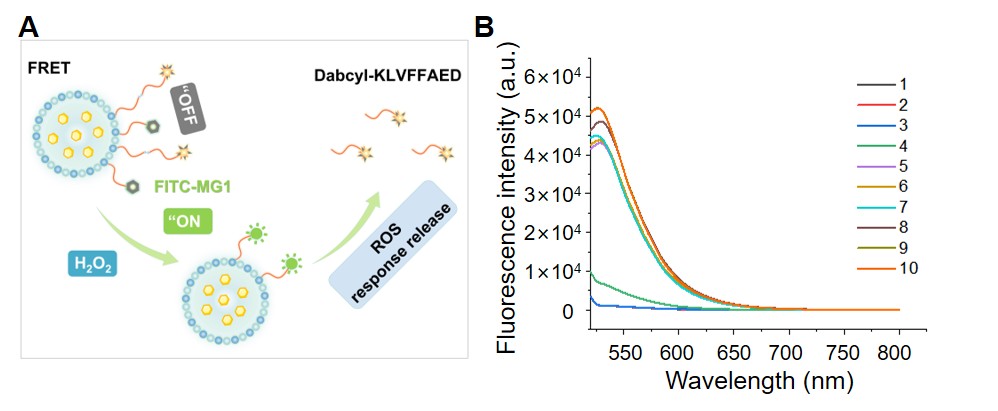


**Fig. S2.** FRET between FITC-MG1 and Dabcyl-KLVFF was used to evaluate the shedding of the KLVFF peptide. (**A**) Illustration of the FRET effect under H_2_O_2_ conditions. (**B**) Changes in fluorescence intensity of FITC under different treatment conditions: (1) MAI@NPs; (2) KMAI@NPs with 0 µM H_2_O_2_; (3) KMAI@NPs with 100 µM H_2_O_2_ for 5 min; (4) ROS-insensitive KMAI@NPs with 100 µM H_2_O_2_ for 5 min; (5) KMAI@NPs with 100 µM H_2_O_2_ for 10 min; (6) ROS-insensitive KMAI@NPs with 100 µM H_2_O_2_ for 10 min; (7) KMAI@NPs with 100 µM H_2_O_2_ for 15 min; (8) ROS-insensitive KMAI@NPs with 100 µM H_2_O_2_ for 15 min; (9) KMAI@NPs with 100 µM H_2_O_2_ for 20 min; (10) ROS-insensitive KMAI@NPs with 100 µM H_2_O_2_ for 20 min.


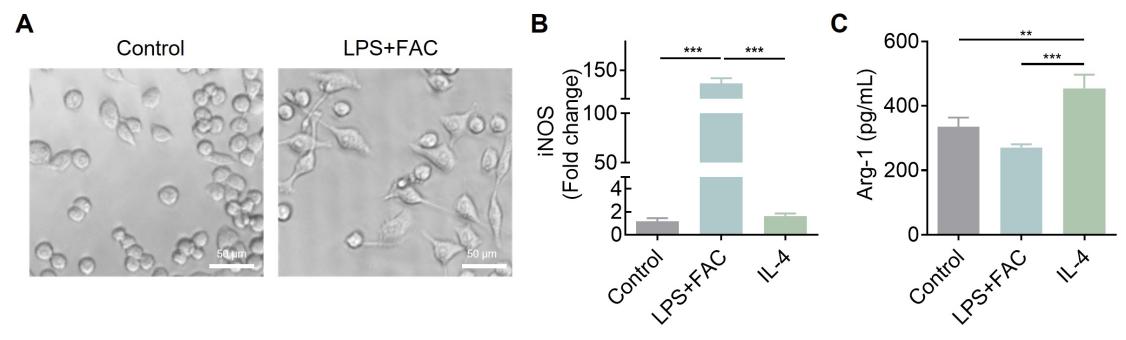


**Fig. S3.** Phenotypic transformation of BV.2 cells. (**A**) Bright-field images of BV.2 cells after treatment with LPS + FAC, captured using an inverted microscope. Scale bar: 50 μm. (**B**) ELISA analysis of the effect of LPS + FAC or IL-4 treatment on iNOS levels in BV.2 cells. (**C**) ELISA analysis of the effect of LPS + FAC or IL-4 treatment on Arg-1 levels in BV.2 cells. Results are reported as mean ± SD (n=3, ^**^*P*<0.01, ^***^*P*<0.001).


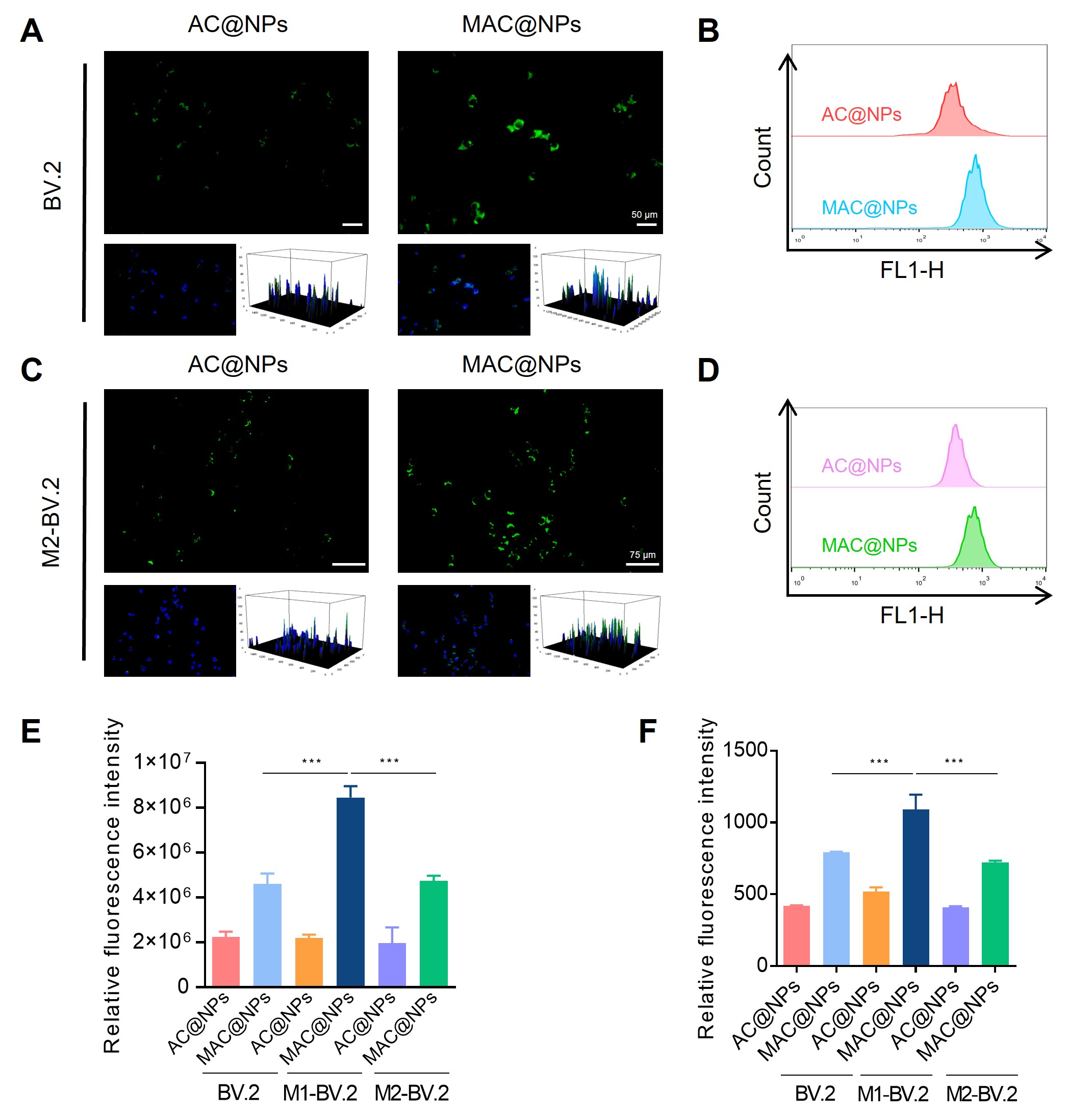


**Fig. S4.** (**A**) Cellular uptake of AC@NPs and MAC@NPs by “resting” microglia, Scale bar: 50 μm. (**B**) Flow cytometry analysis of “resting” microglia uptake of AC@NPs and MAC@NPs. (**C**) Cellular uptake of AC@NPs and MAC@NPs by M2-BV.2 cells, Scale bar: 75 μm. (**D**) Flow cytometry analysis of M2-BV.2 cell uptake of AC@NPs and MAC@NPs. (**E**) Semi-quantitative analysis of fluorescence intensity from microscopy images (A, C). (**F**) Quantitative analysis of fluorescence intensity from flow cytometry data (B, D). Results are reported as mean ± SD (n=3, ^***^*P*<0.001).


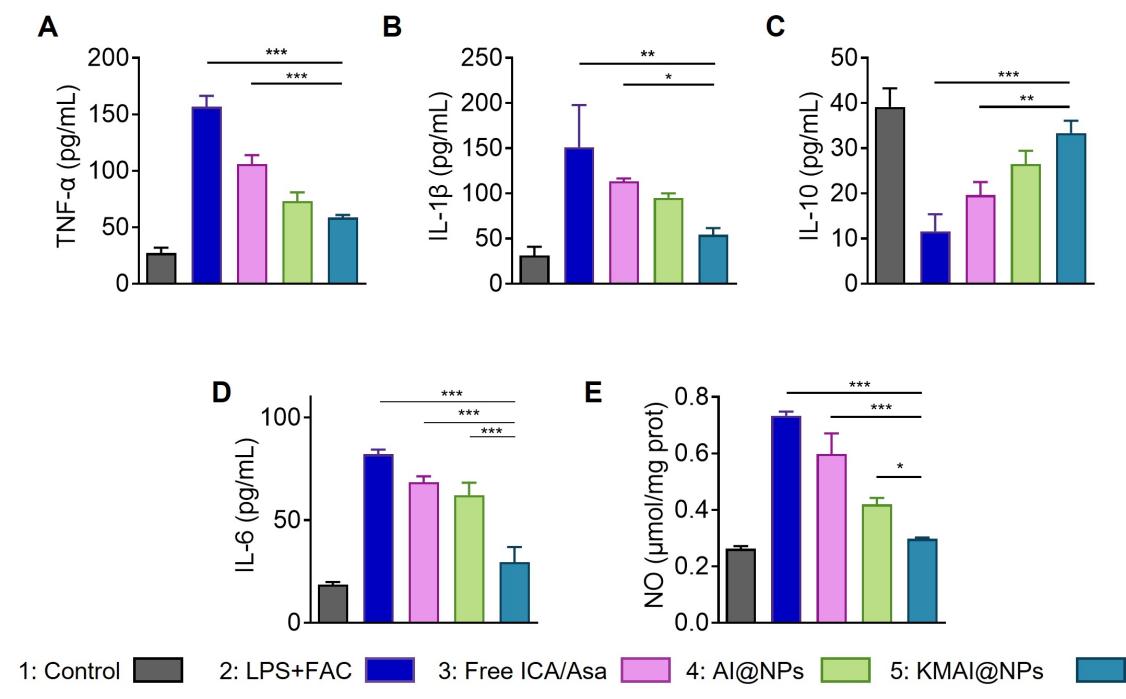


**Fig. S5.** KMAI@NPs modulate the inflammatory secretory profile of activated microglia. (**A–E**) Levels of pro-inflammatory cytokines TNF-α, IL-1β, IL-6, NO, and the anti-inflammatory cytokine IL-10 in the culture supernatants of BV.2 microglial cells following LPS + FAC stimulation and different treatments. Results are reported as mean ± SD (n=3, ^*^*P*<0.05, ^**^*P*<0.01, ^***^*P*<0.001).


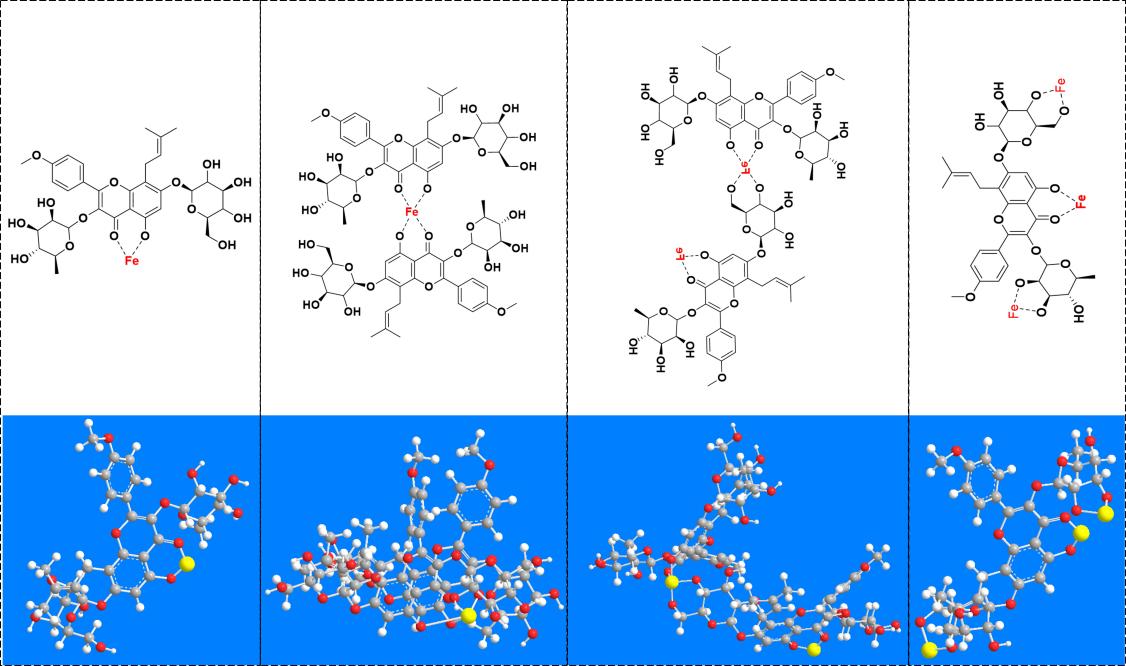


**Fig. S6.** Possible binding sites of iron ions (in ferrous or ferric state) on ICA

**Table S1.** Self-assembly properties of Asa-Dex conjugates synthesized under different conditions

| **Entry** | **Asa (mmol)** | **Dex (Da)** | **Asa conjugation content (%)** | **CAC (mg/mL)** |
| --- | --- | --- | --- | --- |
| 1 | 0.5 | 1000 | 12.2% | Not detected^1^ |
| 2 | 0.5 | 2000 | 9.4% | 0.879 mg/mL |
| 3 | 0.5 | 6000 | 7.7% | 0.0126 mg/mL |
| 4 | 0.01 | 6000 | 0.51% | 0.539 mg/mL |
| 5 | 0.05 | 6000 | 0.63% | 0.277 mg/mL |
| 6 | 0.1 | 6000 | 2.3% | 0.196 mg/mL |
| 7 | 0.25 | 6000 | 3.6% | 0.106 mg/mL |
| 8 | 0.75 | 6000 | 8.5% | 0.0937 mg/mL |
| 9 | 1 | 6000 | 10.3% | 0.691 mg/mL |

^1^ No distinct CAC was detected, which can be attributed to the poor water solubility of the conjugate. As a result, the formation of stable micellar assemblies was hindered.

**Table S2.** Encapsulation efficiency of different NPs (n=3)

| **Groups** | **0 d** | **2 d** | **4 d** | **8 d** |
| --- | --- | --- | --- | --- |
| AI@NPs | (95.986 ±  3.607)% | (91.074 ±  1.803)% | (86.318 ±  2.223)% | (82.597 ±  1.908)% |
| KMAI@NPs | (96.033 ±  3.579)% | (91.473 ±  0.881)% | (88.042 ±  2.591)% | (83.766 ±  2.016)% |

**Table S3.** Hematology data obtained from the mice after various treatments (n=3)

| **Items** | **Wild** | **AD** | **KMAI@NPs** | **Reference range** |
| --- | --- | --- | --- | --- |
| WBC (White Blood Cells)*10^9^/L | 3.6 | 9.8 | 5.1 | 2.5-15 |
| LYM# (Lymphocytes Count)*10^9^/L | 1.85 | 5.34 | 3.25 | 0-15 |
| MON# (Monocytes Count)*10^9^/L | 0.91 | 2.06 | 1.67 | 0-15 |
| GRA# (Granulocytes Count)*10^9^/L | 0.84 | 2.39 | 1.58 | 0-15 |
| LYM% (Lymphocytes Percentage)% | 51.3 | 54.5 | 50 | 0-99.9 |
| MON% (Monocytes Percentage)% | 25.5 | 21.1 | 25.7 | 0-99.9 |
| GRA% (Granulocytes Percentage)% | 23.2 | 24.4 | 24.3 | 0-99.9 |
| HGB (Hemoglobin)g/L | 155.7 | 150.9 | 152.3 | 110-170 |
| MCH (Mean Corpuscular Hemoglobin)Pg | 18.2 | 17.3 | 18.1 | 14-20 |
| MCHC (MCH Concentration)g/L | 296 | 252 | 291 | 250-350 |
| RBC (Red Blood Cells)*10^12^/L | 11.12 | 11.06 | 11.14 | 6.5-12.5 |
| RDW-SD (Red Cell Distribution Width-SD)fL | 26 | 29 | 27 | 0-99 |
| RDW-CV (Red Cell Distribution Width-CV)% | 10.5 | 10.5 | 10.8 | 0-99.9 |
| PLT (Platelets)*10^9^/L | 551 | 546 | 540 | 600-1500 |
| PCT (Plateletcrit)% | 0.522 | 0.532 | 0.52 | 0-0.999 |
| MPV (Mean Platelet Volume)fL | 9.4 | 9.7 | 9.2 | 0-99.9 |
| PDW (Platelet Distribution Width)fL | 11.2 | 12.8 | 11.2 | 0-99.9 |
| P-LCR (Platelet Large Cell Ratio)% | 15.7 | 18.6 | 15.2 | 0-99.9 |

**Table S4.** Summary of molecular docking parameters

| **Compound** | **Target Protein** | **Binding Energy**  **(ΔG, kcal/mol)** | **Ki** | **Hydrogen-Bonding Residues**  **(Distance, Å)** | **van der Waals/H-Bond Energy**  **(kcal/mol)** | **Electrostatic Energy**  **(kcal/mol)** |
| --- | --- | --- | --- | --- | --- | --- |
| ICA | Nrf2 | -7.43 | 3.61 μM | Gly-371 (2.0, 2.7), Val-369 (2.1), Val-608 (2.5), Asp-422 (2.3), Arg-470 (2.7), Asn-469 (1.8), Val-467 (2.0) | -12.24 | -0.26 |
| ICA | GPX4 | -7.3 | 4.43 μM | Asn-28 (1.9, 2.3), Met-26 (2.1), Arg-33 (2.1), Lys-31 (2.5, 2.6) | −11.25 | −0.72 |

**Materials and methods**

**1 Materials, cells, and animals**

Dextran (Mv: 1000~6000 Da): Purchased from Energy Chemical (Shanghai, China). Acetylsalicylic acid (Aspirin, Asa), Icariin (ICA), 4’,6-Diamidino-2-phenylindole (DAPI), 4-Dimethylaminopyridine (DMAP), 1-Ethyl-3-(3-dimethylaminopropyl)carbodiimide Hydrochloride (EDC·HCl), FAC, IL-4, ML385: Purchased from Aladdin Biochemical Technology Co., Ltd. (Shanghai, China). Coumarin, 1,1-Dioctadecyl-3,3,3,3-tetramethylindotricarbocyanine iodide (DiR), Hematoxylin and Eosin (HE) Staining Kit, Nissl Staining Kit, Penicillin-Streptomycin (P/S): Purchased from Meilun Biotechnology Co., Ltd. (Dalian, China). Lipopolysaccharide (LPS): Purchased from Sigma-Aldrich (USA). BCA Protein Assay Kit, Mitochondrial Membrane Potential Assay Kit, CCK-8 Cell Viability Assay Kit, Tissue Iron Content Assay Kit, Cell Iron Content Assay Kit, Micro NO Content Assay Kit, Reactive Oxygen Species (ROS) Assay Kit, Malondialdehyde (MDA), Superoxide Dismutase (SOD), Glutathione (GSH) Assay Kits, Enzyme-Linked Immunosorbent Assay (ELISA) Kits (including Interleukin-6 (IL-6), IL-1β, IL-10 and Tumor Necrosis Factor-α (TNF-α)): Purchased from Solarbio Biotechnology Co., Ltd. (Beijing, China). DSPE-PEG_2000_-TK-PEG_2000_-KLVFF, DSPE-PEG_2000_-MG1: Purchased from Xi’an Ruixi Biological Technology Co., Ltd. (Xi’an, China). Nrf2, Arg-1, iNOS, FTH1, GPX4, BDNF, COX-2, NF-κB, p-NF-κB, TGF-β, GFAP, beta Actin Antibodies, and Horseradish Peroxidase (HRP)-conjugated Secondary Antibodies: Purchased from Affinity Biosciences Co., Ltd. (Jiangsu, China). Aβ_1-42_ Antibody: Purchased from Boster Biological Technology Co., Ltd. (Wuhan, China). Iba-1 Antibody: Purchased from FUJIFILM Wako Pure Chemical Corporation (Osaka, Japan).

**2 Characterization**

^1^H NMR Spectroscopy: The ^1^H NMR spectra were recorded using a Bruker AV-500 or Bruker AV-400 NMR spectrometer. The samples were dissolved in DMSO-*d*_6_ for analysis. Fourier Transform Infrared Spectroscopy (FTIR): FTIR spectra were obtained using a Bruker Equinox 55 FTIR spectrometer (Bruker, Germany) in the range of 400–4000 cm^-1^. Samples were prepared by spreading them on KBr discs and drying them under infrared lamps for 2 min. Each spectrum was recorded by averaging 32 scans at a resolution of 4 cm^-1^ to enhance the signal-to-noise ratio and accuracy. UV-Visible spectra of the samples were measured using a UV-Visible Spectrophotometer U-3010 (Hitachi, Japan). Morphological changes before and after self-assembly: The morphological characteristics of dextran and KMAI@NPs were observed using a Scanning Electron Microscope (SEM, Regulus8100, Hitachi, Japan). The surface morphology of KMAI@NPs was characterized by Transmission Electron Microscopy (TEM, JEM-1200EX, JEOL, Tokyo, Japan). The hydrodynamic diameter, zeta potential, and polydispersity index (PDI) of nanoparticles were analyzed by Dynamic Light Scattering (DLS, Zetasizer Nano ZS90, Malvern, UK). High-Performance Liquid Chromatography (HPLC) equipped with a Diamonsil C18 column (5 μm, 250 × 4.6 mm) was used for the analysis. The mobile phase consisted of acetonitrile: water (25:75 v/v) at a flow rate of 1 mL/min. A UV detector was employed to measure the content of ICA at 275 nm. Flow cytometry analysis was performed using a flow cytometer (BD Biosciences, NJ, USA). Fluorescence microscopy was performed using a Nikon Eclipse E800 microscope (Nikon, Tokyo, Japan) for sample observation.

**3 Preparation of Asa-Dex, DSPE-PEG_2000_-TK-PEG_2000_-KLVFFAED, DSPE-PEG_2000_-MG1, Nanoparticles (NPs)**

Asa-Dex conjugates were synthesized via carbodiimide-mediated esterification, with the feed ratios of Asa to dextran systematically varied to optimize the self-assembly properties of the resulting conjugates. Briefly, a 100 mL three-neck round-bottom flask was placed on a magnetic stirrer under a nitrogen atmosphere. Asa, 4-dimethylaminopyridine (DMAP), and 1-ethyl-3-(3-dimethylaminopropyl)carbodiimide hydrochloride (EDC·HCl) were sequentially added to the flask in predetermined amounts according to the experimental design (Table S1). Anhydrous DMSO (10 mL) was then introduced as the reaction solvent, and the mixture was stirred at 500 rpm at room temperature for 0.5 h to activate the carboxyl groups of Asa. Dextran (400 mg) was dissolved in anhydrous DMSO (10 mL) using an ultrasonic bath to ensure complete dissolution. The dextran solution was subsequently added dropwise to the activated Asa solution, and the reaction was allowed to proceed for 48 h at room temperature under continuous stirring. Upon completion of the reaction, excess ethanol (10-fold the reaction volume) was added to precipitate the crude product. The mixture was allowed to stand overnight, after which the precipitate was collected by filtration or centrifugation. The collected product was transferred to a dialysis bag (molecular weight cutoff: 1000 Da) and dialyzed against distilled water for 48 h to remove unreacted reagents and by-products. Finally, the purified Asa-Dex conjugates were obtained by freeze-drying.

To a solution of 100 mg of DSPE-PEG_2000_-NHS in 5 mL of CHCl_3_, KLVFFAED-PEG_2000_-TK-NH_2_ (1.0 eq.) and Et_3_N (3.0 eq.) were added. The mixture was stirred at room temperature for 0.5 h to ensure complete dissolution and reaction. Afterward, the reaction mixture was concentrated under reduced pressure using a rotary evaporator. The concentrated solution was then precipitated with a large volume of ice-cold ether. The resulting precipitate was collected by filtration and vacuum-dried to yield DSPE-PEG_2000_-TK-PEG_2000_-KLVFFAED.

To a solution of 100 mg of DSPE-PEG_2000_-NHS in 3 mL of DMF, the MG1 peptide (1.1 eq.) and Et_3_N (3.0 eq.) were added. The mixture was stirred at room temperature for 12 h to ensure a complete reaction. After the reaction, the mixture was transferred to a dialysis bag (molecular weight cutoff 2000 Da) and dialyzed against pure water for 24 h. The dialysis solution was then collected and freeze-dried to obtain the DSPE-PEG_2000_-MG1.

KMAI@NPs were prepared by dissolving Asa-Dex (20 mg) in PBS (5 mL), followed by the addition of an ethanol solution containing icariin (ICA, 2 mg), DSPE-PEG_2000_-TK-PEG_2000_-KLVFFAED (4 mg), and DSPE-PEG_2000_-MG1 (4 mg). The mixture was gently shaken to ensure uniform dispersion and subsequently subjected to ultrasonic treatment at 200 W for 10 min to facilitate hydration and homogeneous self-assembly. Ethanol was then removed by rotary evaporation under reduced pressure. To obtain uniform nanoparticles, the resulting dispersion was extruded twice through a polycarbonate membrane with a pore size of 0.22 µm. Nanoparticles without targeting ligands (AI@NPs), as well as nanoparticles modified with a single targeting peptide, including MG1-modified nanoparticles (MAI@NPs), were prepared using the same procedure by omitting or selectively adding the corresponding DSPE-PEG derivatives while keeping the total lipid content constant. To prepare fluorescent probe-loaded nanoparticles, icariin was replaced with coumarin or DiR following the same formulation strategy, yielding AC@NPs, MAC@NPs, and KMAC@NPs (coumarin-loaded), as well as AD@NPs, MAD@NPs@NPs, and KMAD@NPs@NPs (DiR-loaded), respectively. All other preparation conditions were kept identical to ensure comparability among different nanoparticle formulations.

FITC-labeled MG1 peptide and Dabcyl-labeled KLVFF peptide were utilized to modify the nanoparticles, and the peptide loading efficiency on the nanoparticles was quantified using a fluorescence microplate reader. The fluorescence resonance energy transfer (FRET) effect was employed to evaluate the detachment of the KLVFF peptide under H_2_O_2_ conditions by monitoring the changes in fluorescence intensity.

To evaluate the ROS-responsive behavior of KMAI@NPs, 100 μM H_2_O_2_ was added to the nanoparticle solution and incubated for 1 h. The changes in particle size, polydispersity index (PDI), and zeta potential were subsequently measured.

**4 Critical aggregation concentration (CAC) determination**

Due to its high sensitivity to environmental polarity, pyrene was selected as a fluorescent probe to determine the CAC of NPs. A pyrene stock solution (6 × 10^-4^ M in acetone) was prepared, and 4 µL was added to an empty vial. The acetone was allowed to evaporate overnight to eliminate potential interference with NPs formation. Subsequently, 400 µL of NPs solutions at varying concentrations were added to the vial, and the mixtures were gently stirred overnight at room temperature to ensure thorough incorporation of pyrene into the NPs. Fluorescence spectra were recorded with an excitation wavelength of 340 nm and an emission range of 300–400 nm. The fluorescence intensities at 372 nm (I_1_) and 384 nm (I_3_) were measured to calculate the I_1_/I_3_ ratio, which accurately reflects the hydrophobic characteristics of the nanoparticle core. A plot of I_1_/I_3_ ratio versus NPs concentration was constructed, and the CAC was identified as the intersection of two linear regions in the plot, representing the critical point at which NPs formation begins.

**5 Storage stability studies**

The storage stability of KMAI@NPs was evaluated by storing the formulation at 4°C for 8 days. Particle size, PDI, and encapsulation efficiency (EE) were measured on days 2, 4, and 8 to monitor changes over time.

**6 Colocalization of fluorescently labeled NPs with cells**

Based on previous reports and our preliminary experiments, to evaluate the targeted uptake of nanoparticles by M1 microglia, BV.2 cells were stimulated with lipopolysaccharide (LPS, 1 μg/mL) and ferric ammonium citrate (FAC, 200 μM) to induce M1 polarization, with untreated BV.2 cells serving as controls. Cells in different treatment groups were incubated with coumarin-labeled AC@NPs, MAC@NPs, KMAC@NPs, or ROS-insensitive KMAC@NPs for 2 h. Uptake was analyzed using fluorescence microscopy and flow cytometry. Additionally, an AD injury model was established by inducing Bend.3 cells with Aβ_1-42_ (20 μM). These cells were treated with MAC@NPs and KMAC@NPs for 2 h, followed by fluorescence microscopy and flow cytometry analysis (coumarin concentration: 1.5 μM). To evaluate the NPs transport across the BBB in vitro, a BBB model was constructed. AD-Bend.3 cells were seeded at a density of 2 × 10^4^ cells per well in the upper chamber of a Transwell 24-well plate and cultured at 37°C until full confluence. BV.2 cells were seeded in the lower chamber at the same density. After induced M1 polarization of BV.2 cells, fresh culture media containing AC@NPs, KMAC@NPs, or ROS-insensitive KMAC@NPs were added to the upper chamber. After incubation, the medium was removed, and the cells were washed three times with PBS. Fluorescence intensity was qualitatively and quantitatively analyzed using flow cytometry and fluorescence microscopy.

**7 Dose determination for in vitro and in vivo studies**

The dosing regimens for both in vitro and in vivo experiments were determined based on previous literature reports and our preliminary studies. The concentrations used in cell-based assays were selected to ensure sufficient biological efficacy while avoiding cytotoxicity, whereas the doses applied in animal studies were chosen to achieve therapeutic relevance without inducing observable systemic toxicity. For NPs, the actual amounts of ICA and Asa were determined experimentally. The content of Asa in Asa-Dex conjugates was quantified by UV-vis spectroscopy using a calibration curve of free Asa, whereas the encapsulated ICA content was determined by HPLC after separation of free drug, allowing calculation of the encapsulation efficiency and drug-loading content. For comparative studies, the administration doses of free ICA and Asa were calculated to be equivalent to the amounts of ICA and Asa loaded in KMAI@NPs, respectively.

$$\text{Asa}\text{ }\text{conjugation}\text{ }\text{content}\text{ (\%)}\text{=}\frac{\text{C}\text{×}\text{V}\text{×}\text{M}_{\text{Asa}}}{\text{m}}\text{×}\text{100\%}$$

Where *C* is the concentration of Asa, *V* is the solution volume, *M_Asa_* is the molar mass of Asa, and *m* is the mass of the Asa-Dex conjugate.

$$\text{Encapsulation}\text{ }\text{Efficiency}\text{ (}\text{EE}\text{\%)=}\frac{\text{W}_{\text{total}\text{ }\text{ICA}}\text{−}\text{W}_{\text{free}\text{ }\text{ICA}}}{\text{W}_{\text{total}}}\text{×100\%}$$

**8 Evaluation of microglial polarization**

The polarization of microglia was assessed using immunofluorescence staining. BV.2 cells were seeded into 6-well plates at a density of 1 × 10^4^ cells per well and incubated with LPS + FAC for 24 h to induce M1 polarization. After induction, the cells were washed with PBS and treated with media containing PBS, ICA/Asa (free drug combination), AI@NPs, or KMAI@NPs. Following 36 h of incubation, the cells were washed with PBS, fixed with 4% paraformaldehyde for 15 min at room temperature, permeabilized with 0.3% Triton X-100 for 10 min, and blocked with immunostaining blocking buffer for 1 h at room temperature. Subsequently, the cells were incubated overnight at 4°C with primary antibodies, including anti-CD206 and anti-iNOS. After three PBS washes, the cells were incubated with Cy3-conjugated secondary antibody for CD206 staining and FITC-conjugated secondary antibody for iNOS staining for 1 h at room temperature. Nuclei were counterstained with DAPI, followed by PBS washing and imaging using a fluorescence microscope. Cell culture supernatants from different treatment groups were collected to measure pro-inflammatory cytokines (IL-6, TNF-α, IL-1β), anti-inflammatory markers (IL-10), and NO.

**9 Study on the chelation properties of ICA with iron ions**

The chelation properties of ICA with iron ions were investigated using UV-visible spectroscopy. A freshly prepared ICA solution (86 μmol/L, dissolved in 5% DMSO) was mixed with freshly prepared Fe^3^⁺/Fe^2^⁺ solutions (dissolved in deionized water) at a volume ratio of 1:1. The UV-vis absorption spectra of the mixtures were recorded over the wavelength range of 200−500 nm. Changes in the absorption peaks were analyzed to characterize the chelation behavior of ICA with iron ions.

**10 Iron content determination**

Iron levels were quantified using a commercial iron assay kit according to the manufacturer’s instructions. For in vitro experiments, BV.2 cells were seeded in 6-well plates at a density of 1×10^6^ cells/well and cultured overnight. A cellular damage model was established using LPS + FAC. Cells were treated with ICA/Asa, AI@NPs, or KMAI@NPs for 36 h. After treatment, cells were washed twice with cold PBS and lysed in lysis buffer. The lysates were incubated in a shaking incubator for 2 h before further processing. For in vivo studies, fresh brain tissue samples were collected and homogenized on dry ice. The homogenates were incubated in a shaking incubator with lysis buffer for 2 h. The samples were then incubated with 4.5% potassium permanganate and an incubation buffer at 60°C for 1 h, followed by treatment with 30 μL of the iron detection reagent for 30 min. Absorbance was measured at 550 nm using a microplate reader. Protein concentration in each sample was quantified using the BCA protein assay, and the iron content was normalized to protein levels.

**11 Cell viability assay**

HT-22, Bend.3, BV.2, and Neuro-2a cells were seeded in 96-well plates at a density of 5×10^4^ cells/well and cultured overnight at 37°C in a humidified atmosphere with 5% CO_2_ to allow cell adhesion. The cells were treated with Free ICA or KMAI@NPs (*C*_ICA_ : 20 μmol/L) for 48 h. Cell viability was assessed using the CCK-8 assay, with absorbance measured at 450 nm using a microplate reader.

BV.2 cells were seeded and cultured overnight, then treated with Asa-Dex, ICA, AI@NPs, or KMAI@NPs for 4 h the next day. Post-treatment, the cells were extensively washed with PBS to remove residual compounds, followed by exposure to 100 μM H_2_O_2_ for 10 h to induce oxidative stress. Cell viability was quantified using the CCK-8 assay, with six replicates per group.

**12 Intracellular ROS levels**

Intracellular ROS levels were quantified using the DCFH-DA fluorescent probe. BV.2 cells were seeded in 6-well plates and cultured until 90% confluence. Oxidative stress was induced by LPS + FAC, and cells were then treated with ICA/Asa, AI@NPs, or KMAI@NPs. After the treatments, cells were thoroughly washed with PBS and incubated with 10 μM DCFH-DA in the dark for 20 min. The excess probe was removed by additional PBS washes. Fluorescence images were captured using a fluorescence microscope (excitation at 488 nm, emission at 525 nm), and ROS levels were quantified by analyzing the fluorescence intensity with Image J software.

**13 Mitochondrial morphology and function assessment**

The mitochondrial membrane potential was assessed using the fluorescent probe Tetramethylrhodamine Ethylester (TMRE). Cells were cultured and treated with pharmaceuticals as described previously. Post-staining, cellular fluorescence images were captured using a fluorescence microscope to evaluate changes in mitochondrial function. Brain tissue sections were fixed in 2.5% glutaraldehyde for 2 h, dehydrated, dried, and sectioned into ultrathin slices. The slices were stained with uranyl acetate and lead citrate for 15 min, collected onto copper grids, and examined using a transmission electron microscope.

**14 Hemocompatibility evaluation**

To assess the hemocompatibility of the NPs, their hemolytic effects were evaluated. Red blood cells (RBCs) were washed and resuspended in PBS to prepare a 2% (v/v) RBC suspension. An equal volume of NPs at varying concentrations was added, and the mixture was incubated at 37°C for 2 h. RBCs treated with an equal volume of pure water and physiological saline served as positive and negative controls, respectively. The absorbance of the supernatant was measured at 540 nm after centrifugation to remove intact RBCs. The percentage of hemolysis was calculated using the following formula:

$$\text{Hemolysis\%=}\frac{\text{(A}_{\text{sample}}\text{−}\text{A}_{\text{negativecontrol}}\text{)}}{\text{(}\text{A}_{\text{positivecontrol}}\text{−}\text{A}_{\text{negativecontrol}}\text{)}}\text{×}\text{100\%}$$

**15 In vivo biosafety**

For the in vivo biosafety assessment, murine blood samples were collected after treatment to analyze serum enzyme levels and routine blood indices, including albumin (ALB), alkaline phosphatase (ALP), alanine aminotransferase (ALT), aspartate aminotransferase (AST), urea (UREA), creatinine (CREA), and uric acid (UA). White blood cell (WBC) counts, as well as lymphocyte (LYM#), monocyte (MON#), and granulocyte (GRA#) counts, and their respective percentages (LYM%, MON%, GRA%), were also determined. Other parameters included hemoglobin (HGB), mean corpuscular hemoglobin (MCH), mean corpuscular hemoglobin concentration (MCHC), red blood cell count (RBC), red cell distribution width (RDW) - standard deviation (RDW-SD) and coefficient of variation (RDW-CV), platelet count (PLT), platelet crit (PCT), mean platelet volume (MPV), platelet distribution width (PDW), and platelet large cell ratio (P-LCR). Major organs (heart, liver, spleen, lungs, and kidneys) were harvested, fixed in 4% paraformaldehyde, and processed for histological sectioning. HE staining was performed, and the resulting sections were examined for pathological changes.

**16 In vivo biodistribution and blood circulation**

To investigate the distribution of different nanoparticles in APP/PS1 mice, physiological saline, free DiR, AD@NPs, MAD@NPs, and KMAD@NPs were administered via tail vein injection. A live fluorescence imaging system (IVScope 8200, Clinx, China) was used to capture fluorescence images of the mice at specified time points (1, 3, 6, 12, 24, 36, 48, and 72 h). At 72 h post-administration, the brain, heart, liver, spleen, lungs, and kidneys were collected for ex vivo fluorescence imaging. To assess blood circulation, blood samples were collected from the APP/PS1 mice at predetermined time points post-injection to measure the fluorescence intensity in the bloodstream.

**17 Nrf2 inhibition assay**

To elucidate the involvement of Nrf2 signaling in the regulatory effects of KMAI@NPs on microglial ferroptosis susceptibility and functional stability, a pharmacological inhibition strategy was employed using the selective Nrf2 inhibitor ML385. Briefly, BV.2 microglial cells were seeded in culture plates and allowed to adhere under standard culture conditions. Cells were pretreated with ML385 to suppress Nrf2 transcriptional activity, followed by stimulation with LPS + FAC to establish an inflammatory and iron-overload microenvironment. Subsequently, cells were treated with KMAI@NPs. After treatment, cells were collected for downstream analyses. Nrf2 activation and subcellular localization were evaluated by immunofluorescence staining and by Western blot analysis. The expression of ferroptosis-related proteins, including GPX4, was assessed by Western blotting. Intracellular iron levels, GSH content, and MDA levels were quantified using commercial assay kits according to the manufacturers’ instructions. In parallel, cytokine secretion profiles were analyzed to assess the functional consequences of Nrf2 inhibition on microglial inflammatory responses.

**18 Molecular docking studies**

To investigate the binding interactions between ICA and key targets (Nrf2, GPX4), molecular docking simulations were performed using AutoDockTools-1.5.7 (ADT) and AutoDock Vina 1.2.0. The crystal structures of human Nrf2, GPX4 were obtained from the RCSB Protein Data Bank. Water molecules and non-essential cofactors were removed using ADT, and polar hydrogen atoms were added with Kollman charges assigned. The 3D structures of ICA was downloaded from PubChem, energy minimization was performed using the UFF force field in Open Babel, and the structures were converted to PDBQT format using ADT. Next, grid box configurations were set up for docking. The docking simulations were carried out using the Lamarckian genetic algorithm with the following parameters: population size: 150, maximum energy evaluations: 2,500,000, number of genetic algorithm runs: 50, exhaustiveness: 8, with other parameters set to default values. Finally, validation and analysis were performed, including re-docking of the co-crystallized ligands (RMSD < 2.0 Å) to confirm the reliability of the protocol. The highest-scoring conformations (lowest binding energy, ΔG) were selected for each complex. Hydrogen bonds, hydrophobic interactions, and π-π stacking were visualized using PyMOL.

**19 Western blotting analysis**

Proteins were extracted from BV.2 cells and brain tissue. The expression levels of GPX4, Nrf2, FTH1, Arg-1, TGF-β, iNOS, NF-κB, COX-2, Aβ, and BDNF were assessed. Protein concentrations were measured using the BCA assay. Proteins were separated by SDS-PAGE and transferred to a polyvinylidene fluoride (PVDF) membrane. The PVDF membrane was blocked with a 5% BSA solution for 1 h, followed by overnight incubation at 4°C with primary antibodies. After washing with TBST, the membrane was incubated with HRP-conjugated secondary antibodies at room temperature for 1 h. Following TBST washes, protein bands were detected using the ECL method, and protein expression levels were quantified using Image J software. Comparative analysis of protein expression levels across experimental groups was conducted to assess the impact of each treatment on these critical pathways.

**20 In vivo evaluation of anti-AD efficacy**

All mice used in this study were purchased from Beijing Huafukang Biotechnology Co., Ltd. The 8-week-old male C57BL/6J wild-type mice and APP/PS1 transgenic male mice were used in accordance with the ethical guidelines approved by the Laboratory Animal Management and Use Committee of Liaoning University of Traditional Chinese Medicine, with ethical approval for animal experiments granted under Approval No. 210000420240205. Mice were randomly assigned to the following experimental groups: wild-type group (C57BL/6J + saline), AD group (APP/PS1 + saline), and treatment groups (5 mg/kg), including free drug group (APP/PS1 + ICA/Asa), AI@NPs group (APP/PS1 + AI@NPs), and KMAI@NPs group (APP/PS1 + KMAI@NPs). Each group consisted of six mice.

After the treatment period, behavioral assessments were conducted for 10 days, including the Morris water maze test and nest-building behavior test, to evaluate cognitive and behavioral function. Upon completion of the behavioral testing, the mice were euthanized, and brain tissues were harvested for pharmacological analysis. Levels of inflammatory cytokines (IL-10, TNF-α) and oxidative stress markers (malondialdehyde, superoxide dismutase, glutathione) were measured in brain tissues to assess the effects of the different treatments on inflammation and oxidative stress in APP/PS1 mice. Transmission electron microscopy (TEM) was employed to observe morphological changes in the Golgi apparatus in brain tissues following treatment. Furthermore, histopathological analysis of paraffin-embedded brain tissue sections was performed using HE staining and Nissl staining to evaluate neuronal damage. Immunohistochemical staining was used to assess the expression of astrocyte marker GFAP, and microglia marker Iba-1, as well as the expression levels of Aβ, FTH1, and GPX4. Immunofluorescence staining was performed to detect the expression levels of Iba-1^+^/iNOS^+^, Iba-1^+^/Arg-1^+^, and Iba-1^+^/Nrf2^+^ in brain tissues.

**21 Statistical Analysis**

All data were analyzed using GraphPad Prism 9 Software and expressed mean ± SD. Significant differences were assessed using Student’s *t* test or one-way analysis of variance (ANOVA) followed by Tukey’s multiple comparisons tests. ^*^*P*<0.05, ^**^*P*<0.01, ^***^*P*<0.001 were determined as statistically significant.
